# Evaluation of Peptide-Based Vaccines Against *Group A Streptococcus* in *Staphylococcus aureus*-Infected Mice

**DOI:** 10.3390/vaccines13060632

**Published:** 2025-06-12

**Authors:** Ahmed O. Shalash, Haolan Sun, Yiru Cui, Jingwen Wang, Barb Arnts, Jannah Bauer, Waleed M. Hussein, Zeinab G. Khalil, Mariusz Skwarczynski, Istvan Toth

**Affiliations:** 1 School of Chemistry and Molecular Biosciences, The University of Queensland, Brisbane, QLD 4072, Australiam.skwarczynski@uq.edu.au (M.S.);; 2 Australian Institute for Bioengineering and Nanotechnology, The University of Queensland, Biological Resources, Brisbane, QLD 4072, Australia; 3 Institute for Molecular Bioscience, The University of Queensland, Brisbane, QLD 4072, Australia; 4 School of Pharmacy and Pharmaceutical Sciences, The University of Queensland, Brisbane, QLD 4072, Australia

**Keywords:** *Group A Streptococcus*, vaccines, immunogenicity, immune response quality, novel antigens, Mutant bacterial strains, *S. aureus*-infected mice

## Abstract

Background: *Group A Streptococcus* (GAS) is a major human pathogen associated with serious diseases. Evaluating immune responses against GAS vaccines—immunogenicity, quality, and efficacy—is complicated by interference from co-infections, like *Staphylococcus aureus* (*S. aureus*). We aimed to evaluate peptide-based GAS vaccines in mice for antisera efficacy against standard and mutant GAS strains and to assess immunological methods under co-infection conditions. Methods: Female C57BL/6 mice were infected with *S. aureus* and immunized with various M-protein-derived peptide antigens: J8, J8i, J8i-J8i, and the native p145 sequence. Two novel, conserved M-protein-derived antigens (NTD and CTD2) were also evaluated. Enzyme-linked immunosorbent assays (ELISAs) were used to assess immunogenicity and GAS-specific antibody responses. Peptide antigens were either conjugated to or physically mixed with the PADRE T-helper epitope and tested for enhanced antisera immunogenicity and opsonic efficacy. Result: ELISA against the immunizing peptides as coating antigens reflected the immunogenicity, while p145-based ELISA correlated with GAS-specific antibody titres without *S. aureus* interference for J8-based vaccines. Immunogenicity ranked J8 > J8i ≈ J8i-J8i > p145. NTD and CTD2 antisera demonstrated opsonic activity, indicating protective potential. PADRE–J8 conjugates significantly enhanced antibody magnitude and quality, producing strong opsonic bactericidal responses against both standard and p145-mutant GAS strains. PADRE–J8i was effective only against standard strains. This is the first report to suggest at least two B-cell epitopes within the J8i peptide. Conclusion: These findings support the diagnostic utility of p145, NTD, and CTD2 under co-infection settings, and the vaccine potential of J8, NTD, and CTD2, particularly when conjugated to a T helper for enhanced antigen presentation.

## 1. Introduction

*Group A streptococcus* (GAS) infection causes a wide range of human diseases, from mild to severe life-threatening illnesses, like invasive GAS, affecting over 600,000 patients each year, with a 25% mortality rate [1,2,3]. As beta-lactam antibiotics continue to lose their efficacy, GAS infection remain among the top ten infectious diseases causing mortality worldwide, and there is an urgent need to develop an effective vaccine and immunological evaluation methods that are broadly applicable to suit various research settings [2,4,5].

A successful vaccine must induce high-quality, opsonic antibodies capable of recognizing GAS during infection, while immunological assays should reliably measure these responses, even in the context of co-existing or prior infections [3,6,7,8].

**Figure 1:**
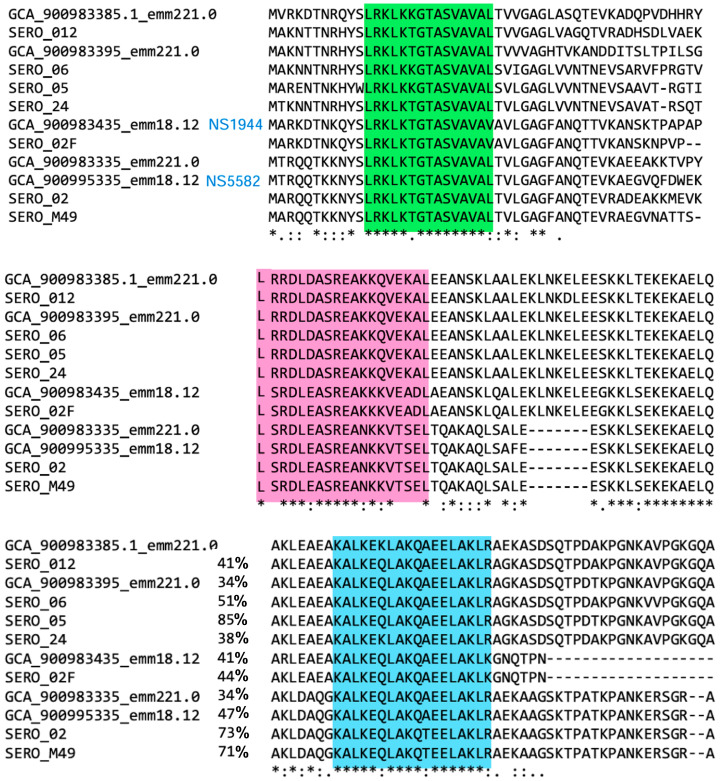
Partial sequences from full-length M-protein alignment using Clustal-w, from different GAS serotypes, including serotypes 2, 2F, 4, 5, 6, 12, 24, M49, M221.0, and M18.12 [GCA_900983435 (NS1944) and GCA_900995335 (NS5582) mutant strains], obtained from Uniprot and the protein database. Derived peptides are NTD (LRKLKKGTASVAVAL; highlighted in green colour, top); p145 (LRRDLDASREAKKQVEKALE, enclosing J8i (SREAKKQVEKAL), highlighted in pink, middle); and CTD2 (KALKEQLAKQAEELAKLR, highlighted in blue, bottom), asterisks represent highly conserved residues among GAS strains.

Among the streptococcal surface antigens, the M-protein is the most prominent vaccine target for GAS vaccine development. The M-protein is a major virulence factor of GAS and a well-characterized cell wall-anchored surface antigen (Figure 1). Structurally, it is an α-helical coiled-coil dimer protein, which is anchored to the bacterial cell wall via a conserved *C*-terminal LP*X*TG motif [9]. The coiled-coil dimeric structure of the M-protein facilitates immune evasion [10]. M-protein further supports host immune modulation by binding to host plasmin(ogen) and fibrinogen, inhibiting complement activation, degrading a key opsonin (C3b), and thus aiding in opsonization and phagocytosis escape, and it plays a role in adhesion to host cell glycans [11,12,13,14].

M-protein’s *N*-terminal region (50 residues) is the emm-defining segment, and it is hypervariable [15]. The *N*-terminal region is immunogenic and surface exposed; however, it is a risky antigen source due to limited strain coverage. In contrast, the *C*-terminal membrane-proximal region is highly conserved, and thus a good source of vaccine antigens with high coverage against many GAS strains. However, full-length M-protein vaccines risk eliciting autoimmune responses due to sequence similarities with human myosin [16,17,18,19]. In addition, with over 250 serotypes exhibiting M-protein sequence variations, the usage of a single homolog of M-protein, 34 to 85% conserved (51.3 ± 17.4 average, Figure 1), or its variable fragments as a vaccine antigen will allow mutant escape and provide limited coverage against diverse GAS strains [3].

To overcome these challenges, two strategies were adopted to develop M-protein-based vaccine antigens against GAS. These strategies aimed to broaden vaccine coverage, to prevent mutant escape, and to avoid eliciting cross-reactive autoimmune responses. These strategies are either using (1) a minimal and conserved *C*-terminal-derived immunogenic peptide antigen (like J8 peptide), or (2) a modified chimeric protein combining multiple strain-specific *N*-terminal fragments (like the StreptAnova^®^ vaccine antigen) [20,21].

The J8 peptide antigen (**QAEDKVKQ**-SREAKKQVEKAL-**KQLEDKVQ**, bold residues are derived from yeast GCN-4 protein [22]) was designed to include a highly conserved sequence; however, its M-protein-derived sequence content is only 58 to 100% conserved (80.3 ± 19.8 average) and reported to be found in only 37% of GAS genomes [3]. J8 was also designed to avoid a shared epitope with human myosin in the native p145 epitope sequence (**LRRDLDA**-SREAKKQVEKALE, bold residues are shared with human myosin, 60 to 100% conserved, 80.0 ± 19.5 average). J8 is also flanked with GCN-4 sequences at both termini of the minimalist epitope J8i (SREAKKQVEKAL, 58 to 100% conserved, 80.3 ± 19.8 average) to impart an equivalent helicity to the p145 original native sequence’s conformation. The J8 peptide is known to induce broadly protective opsonic antibodies. M-protein is completely helical in structure, and thus imparting helicity to M-protein-derived antigens such as J8 in peptide vaccines significantly improves their immunogenicity and efficacy [6,23]. To further assist the presentation of J8 to antigen-presenting cells, various approaches have been adopted, such as (a) conjugation to a carrier protein, like diphtheria toxoid [24], or (b) conjugation of a hydrophobic moiety, with adjuvanting properties, to enable self-assembly into an immunogenic nanovaccine, such self-adjuvanting moieties including polyhydrophobic amino acids, like polyleucine, or lipid core peptides [6,7].

**Figure 2:**
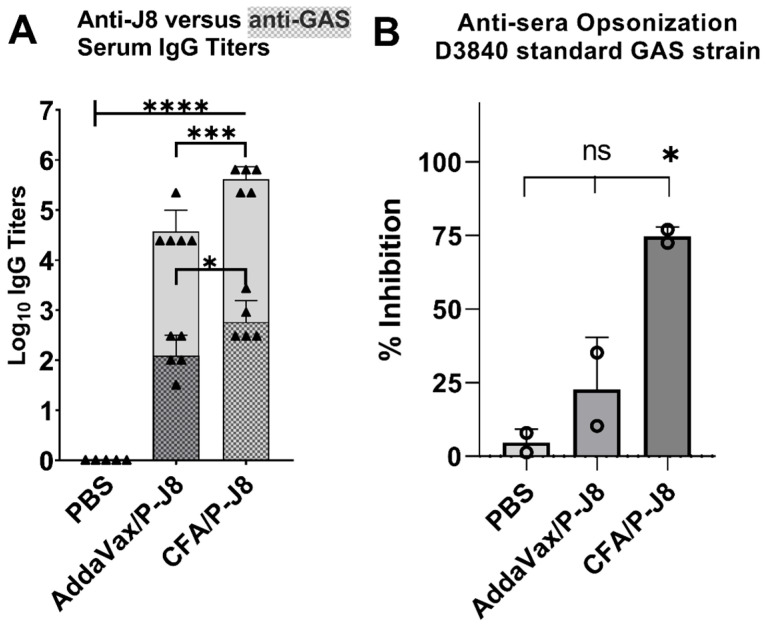
Immune responses and efficacy of J8, adapted from [25]. (**A**) Serum antigen-specific IgG generated against PADRE-J8 (P-J8) peptide antigen in C57BL/6 mice (n = 5), where the shaded portion represents anti-GAS titres (GAS bacteria coated on ELISA plates) compared to the anti-J8 peptide titres, while the gaps represent 300- and 600-fold for AddaVax and CFA vaccines, respectively, between generated anti-peptide titres and anti-GAS high-quality antibodies. (**B**) The opsonic capacity of those 10-fold diluted antisera generated against J8, with a small difference between the two vaccines, resulted in a dramatic change in opsonization bactericidal efficacy. One-way ANOVA with Dunnett’s multiple comparisons test was used for statistical analysis. Significance was set to *p* < 0.05 (*), *p* < 0.001 (***), and *p* < 0.0001 (****).

In contrast, the other approach employs the hypervariable *N*-terminal segment of M-protein, but due to its inter-strain variability, the StreptAnova^®^ vaccine combines 30-valent antigens, each of 50 amino acids, from different GAS strains [20,21], into a single chimeric protein sequence that protects against these strains, imparting broader coverage. However, protection is limited mostly to the 30 GAS strains, from which antigens were derived [8,21]. Therefore, in either strategy, the antigens are intentionally abbreviated or have minimalist immunogenic components to prevent the autoantigen sequences located at the *C*-terminus of M-protein from inciting autoimmune responses, thus preventing the generation of cross-reactive antibodies [8]. Thus, when developing GAS vaccines using such derived and non-native antigen sequences, e.g., GCN-4 sequences or carrier proteins, the quality of the elicited immune responses and the absence of cross-reactivity are constantly being investigated [17,18,19], and further improvements in the quality of immune responses could result in dramatic efficacy improvements (Figure 2) [25]. For example, a significant proportion of generated antibodies against J8 has been hypothesized to be directed against the irrelevant GCN-4 flanking sequences (Figure 2A).

Ongoing research is being conducted to discover novel, highly conserved, and immunogenic peptide antigens, derived from various surface proteins, to be employed in developing next-generation GAS vaccines, like FNBR and S2 [26]. Such antigens could be useful in triggering broader immune responses and reducing mutant escape.

Gram-positive and immobile *Staphylococcus aureus* (*S. aureus*) is a type of cocci bacteria, which infects all mammalian species and is commonly harboured in the nasopharynx of 30% of healthy humans. Human infections cause septicaemias and localised inflammatory diseases, but the majority of infected animals and humans are asymptomatic. Thus, infected immunocompetent mice do not exhibit symptoms of illness. Infection is mucosal and predominantly localizes to the epidermis, gastrointestinal system, and nasopharynx, which are the primary sites of *S. aureus* colonization [27,28]. Further, the infection is easily transmissible via several different routes, including contaminated aerosol or direct contact with fomites, from infected animals, where animals housed/caged together are expected to share the infection through breathing contaminated aerosols or via contact during grooming. Such a common infection will serve to establish potentially interfering or cross-reactive immune responses against GAS that originally result from a pre-existing *S. aureus* infection.

In this study, we aim to (1) discover novel, conserved, and minimalist surface-derived peptide antigens, and (2) establish immunological assays that can evaluate the magnitude, type, and quality of generated antibodies against native streptococcal surface antigens in the presence of a co-infection. We seek develop robust immunological assays in the presence of potential interference from prior or co-existing infections, more similar to clinical settings. Thus, we initially evaluated M-proteins from over 12 diverse GAS strains (Figure 1) and identified two highly conserved sequences, NTD (LRKLKKGTASVAVAL, 87 to 100% conserved, 93.3 ± 4.2 on average) and CTD2 (KALKEQLAKQAEELAKLR, 89 to 100% conserved, 97.0 ± 3.5 on average), compared to J8i or p145. These peptides were selected based on their surface exposure, immunogenic potential, and ability to induce bactericidal antibodies across GAS strains (strain coverage), including those with p145 sequence mutations. To this end, we synthesized J8-derived peptide antigens, including J8, J8i, p145, and J8i-J8i dimer, as well as the new antigens, characterized their secondary structure, and adjuvanted with either complete Freund’s adjuvant (CFA) or 15-mer polyleucine to immunize C57BL/6 mice, which were infected, since birth, with *S. aureus*. We also compared the outcomes to immunized, non-infected mice, with CFA-immunized J8 or saline as controls (Figure 2). We conducted ELISA against each peptide (J8, J8i, J8i-J8i, p145, NTD, and CTD2) and used carvacrol-inactivated whole-GAS bacteria as ELISA coating antigens. Finally, we conducted a bactericidal/opsonization assay for the antisera. Ultimately, the immune responses against each antigen correlated with the recognition of whole GAS bacteria or the overall bactericidal efficacy. In other words, our goal was to investigate which of the ELISA coating antigens (J8, J8i, p145, NTD, CTD2, or whole inactivated GAS) best represented each of the following: (1) the magnitude, (2) quality/avidity, or (3) bactericidal efficacy of generated antigen-specific antibodies against GAS. Moreover, we also evaluated the usefulness of the two newly discovered peptide antigens.

## 2. Materials and Methods

### 2.1. Materials

Analytical-grade reagents were used throughout the experiments. Dichloromethane (DCM), *N*,*N*-dimethylformamide (DMF), chromatography-grade methanol (MeOH) and acetonitrile (MeCN), and peptide-grade piperidine were obtained from Merck (Kilsyth, VIC, Australia). *N*,*N*,*N*′,*N*′-tetramethyl-O-(7-azabenzotriazol-1-yl)uroniumhexafluorophosphate (HATU) was obtained from Mimotopes (Melbourne, VIC, Australia). Trifluoroacetic acid (TFA) and diisopropylethylamine (DIPEA) were acquired from Novachem (Heidelberg West, VIC, Australia). 2,2,2-Trifluoroethanol (TFE) was obtained from ThermoFisher Scientific (Banyo, QLD, Australia). Fluoronylmethoxycarbonyl (FMOC)-protected amino acids and Rink amide resin (100–200 mesh, substitution value of 0.56 mmol/g) were acquired from Novabiochem (Läufelfingen, Switzerland). Horseradish peroxidase (HRP)-goat anti-mouse IgG, phosphate buffer (PBS), O-phenylenediamine dihydrochloride (OPD) substrate, and triisopropylsilane (TIPS) were obtained from Sigma Aldrich (Castle Hill, NSW, Australia). Microplates (96-well, flat base, polystyrene, high binding) were obtained from Sarstedt (Mawson Lakes, SA, Australia).

### 2.2. Equipment

Reverse-phase analytical liquid chromatography (RP)-HPLC was employed for measuring peptide purity using a Shimadzu HPLC system (SPD-M10A VP, LC-20AB, DGU-20A5, SIL-20AC HT, Shimadzu, Kyoto, Japan) equipped with a Grace Vydac 214TP C4 5 μm column or Vydac 218TP C18 5 μm column. The mobile-phase flow rate was set to 1 mL/min with gradient elution using a mixture of solvent A (100% Milli-Q water and 0.1% TFA) and solvent B (90% MeCN in Milli-Q water and 0.1% TFA). Preparative HPLC was employed for peptide purification on a Shimadzu HPLC system (SPD-20A, LC-20AP, CBM-20A, FRC-10A, Shimadzu, Kyoto, Japan) equipped with a C4 10 μm (Grace Vydac, 214TP1022) or C18 10 μm (Grace Vydac, 218TP1022) column. The mobile-phase flow rate was set to 20 mL/min using a gradient of solvents A and B. Synthesized peptide identity was confirmed via electrospray ionization mass spectrometry (ESI-MS) (PerkinElmer-Sciex API3000, Concord, ON, Canada). ESI-MS solvent was a 1:1 mixture of solvent C (100% Milli-Q water and 0.1% AcOH) and solvent D (90% MeCN in Milli-Q water and 0.1% AcOH) and was set to a flow rate of 0.5 mL/min. Particle size distribution of self-assembled peptide vaccines was measured using dynamic light scattering (DLS) (zeta sizer 3000TM, Malvern Instruments, Malvern, UK). The secondary structure of the peptide antigens was analysed using a Jasco-720 spectropolarimeter (Jasco Corp., Tokyo, Japan). Optical density (OD) values of immunological assays were measured by the SpectraMAX 250 plate reader (Molecular Devices, San Jose, CA, USA).

### 2.3. Peptide Synthesis

**Figure 3:**
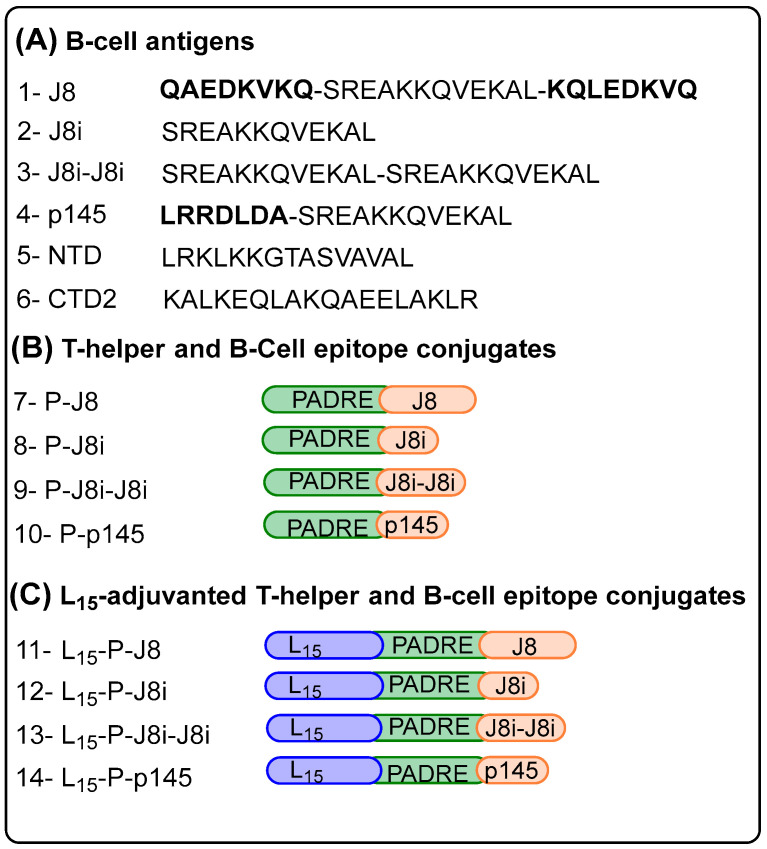
Synthesized peptides: (**A**) B-cell antigens, (**B**) T-helper epitope conjugates with B-cell antigens, and (**C**) 15-mer polyleucine (L_15_, blue oval), T-helper epitopes (PADRE: (P) green oval), and B-cell epitopes (orange oval).

Peptide antigens (Figure 3 and Table 1) were synthesized using standard Fmoc solid-phase peptide synthesis (SPPS) as previously described [6,29]. Briefly, a 0.1 mmole scale of Rink amide resin was weighed for each peptide and allowed to swell in DMF overnight. After swelling, DMF was removed and a solution of 20% piperidine in DMF was added twice (5 min × 2) to remove Fmoc protection groups on the functionalized resin reaction sites. After deprotection of resin, the first C-terminal FMOC-protected amino acid in the sequence was weighed (4.2 equivalents) and activated using HATU (4.0 equivalents) and DIPEA (6.2 equivalents) in DMF, then added to the resin for coupling twice (20 min × 2). The resin was washed five times with DMF and deprotected again as mentioned above, followed by coupling the following fmoc-protected amino acid in the peptide sequence. Cycles of deprotection and coupling were continued till the entire peptide antigen sequence was complete. The peptidyl-resin was deprotected from N-terminal Fmoc once more, washed with DMF (×3), DCM (×3), and MeOH (×3), and left to dry overnight under vacuum at room temperature (RT). Each peptide antigen was cleaved from the resin using a 10 mL mixture of TFA, Milli-Q water, and TIPS (95:2.5:2.5) while stirring (250v RPM) for 4 h at RT. Afterwards, the resin was removed by filtration, and the TFA-containing peptide solution was dried under a vacuum. The dried peptide was washed with ice-cold diethyl ether: *N*-hexane three times, to remove the scavengers. The precipitated peptide antigen was filtered under vacuum, redissolved in a suitable volume of acetonitrile: water (1:1) solution, and purified using preparative HPLC.

### 2.4. Preparation of Vaccines

The vaccines included CFA-adjuvanted peptide antigens or 15-mer polyleucine adjuvanted compounds and were all adjusted to a final antigen concentration of 0.5 mg/mL, to deliver 25 µg per 50 µL dose/mouse. CFA vaccines were prepared by vortexing the adjuvant (bacterium suspension in mineral oil) for 1 min and taking the required volume of adjuvant and mixing 1:1 *v*/*v* with the peptide antigen solution in PBS. Afterwards, CFA emulsification was conducted using an ultrasound probe homogenizer by sonicating at 40% power and 40% pulser for 5 min in an ice bath. Meanwhile, polyleucine compounds were simply dissolved in PBS, to achieve an antigen concentration of 0.5 mg/mL in PBS, and vortexed for 1 min.

### 2.5. Characterization of Vaccines: Size, Shape, and Structural Conformation

The particle size and polydispersity (PDI) of self-assembled polyleucine vaccines were evaluated using DLS at a scattering angle of 173° and 25 °C using disposable cuvettes. The vaccine candidates were dissolved at a concentration of 0.1 mg/mL using PBS, and the size was presented as an average of 5 measurements.

Transmission electron microscopy (TEM) was used to confirm the self-assembled polyleucine vaccines’ sizes and investigate their shapes. The samples (0.1 mg/mL concentration) were dropped onto a carbon-coated grid and were stained using uranyl acetate and dried before imaging via a Joel electron microscope (JEM-1010, JEOL Ltd., Tokyo, Japan) operated at 100 kV.

The secondary structure of the peptide antigens was analysed using circular dichroism. Each peptide was dissolved at a concentration of 0.25 mg/mL in 10 mM PBS, a volume of 200 µL was added to a 1 mm quartz microcuvette, and we performed measurements, then a further 25% TFE was added, mixed well, and remeasured. Quantitative secondary structural determination was performed on the resulting molar ellipticity data, where the resulting composite spectra were fitted to yield the contribution of each pure spectral component, i.e., β-sheet, random coil, or α-helix spectrum. The individual pure spectra were previously reported using polylysine peptide [30], and the quantitative secondary structure was determined as described previously [6].

### 2.6. In Vivo Assessment in Mice

The mice were obtained from Australian Bio Resources (ABR, New South Wales, Australia), and animal experimentation procedures were approved by the University of Queensland Animal Ethics Committee (AEC), ethics number 2021/AE000311. Naïve (uninfected) or *S. aureus*-infected C57BL/6 female mice (6–8 weeks old) were randomly housed in groups of four. After acclimatization for a week, pre-immunization, 10 μL of blood was collected from each mouse two days before immunization (-2d) from the tail tip and diluted 10-fold in PBS. CFA-immunized mice were subcutaneously immunized, and blood was collected 42 days post-immunization (42d) after euthanasia. Polyleucine vaccine-immunized mice were immunized on days 0, 14, and 28, then sera were collected 42 days post-immunization after euthanasia. Mice were generally euthanized using CO_2_ on the 42nd day post-immunization, and blood was collected via heart puncture. The collected blood (undiluted or diluted) was centrifuged at 3600 rpm for 10 min, and sera were transferred and stored in centrifuge tubes at −80 °C for further immunological analyses.

Mice were infected since birth by *S. aureus*, and infection was confirmed by capillary electrophoresis. Faecal extracts were tested for *Staphylococcus aureus* through 3 different PCR primer pairs that specifically target the *S. aureus* DNA sequence of the Sa422, GyrA, and Spa genes, while excluding the DNA sequence of other bacterial pathogens. This ensured that all *S. aureus* species would be detected if present; it also allowed for any false positives to be identified and ruled out. The testing was performed on faecal samples pooled from each cage. Bacterial DNA extraction was performed using a Qiagen QIAamp Power Fecal Pro extraction kit. Extracts were tested with generic bacterial 16S primers to verify successful extraction and that the negative results were valid. The testing was performed using fluorescently labelled PCR primers, and then the PCR products were detected via capillary electrophoresis on an Applied Biosystems 3730 DNA analyser. The results were then viewed as fluorescence chromatograms in specialised software. Initial positive PCR results, as well as positive PCR results from a ZymoBIOMICS Microbial Community Standard control (including *S. aureus*), were also sequenced by Sanger sequencing to validate the test results.

### 2.7. Evaluation of Serological Immune Responses: Immunogenicity (Against Peptides), Avidity (Against GAS), and the Cross-Reactivity Between the Two Bacteria

The enzyme-linked immunosorbent assay (ELISA) was performed to determine the antigen-specific IgG antibody titre. Coating was performed using each peptide antigen or whole inactivated GAS bacteria, as described previously [31], to determine the magnitude, type, quality, or potential cross-reactive immune responses. To this end, serum antibody titres against the same peptide antigen used in vaccination represented immunogenicity and/or type of triggered immune response. Meanwhile, serum antibody titres against native M-protein derived sequences (p145, J8i, NTD, and CTD2) and against whole GAS bacteria represented immune responses of high-quality and cross-reactive ones between GAS and *S. aureus*. Microplates (96-well) were coated with peptide antigen at a concentration of 0.3 μg/well by dissolving peptide in a pH 9.6 carbonate coating buffer (CCB) and incubating at 37 °C for 90 min. Alternatively, for a whole inactivated GAS bacterium coating, bacterium suspension at a concentration of 2.5 × 10^8^ CFU/mL in CCB was added to each well and left to bind overnight at 4 °C. The plate contents were discarded and 250 μL/well of 5% *w*/*v* skim milk solution was added and incubated at 37 °C for 90 min, then plates were washed. Serum samples were added to the first row at a 1:100 final dilution and 3-fold serially diluted down the columns in 0.5% *w*/*v* skim milk solution. and plates were incubated at 37 °C for 90 min followed by washing. HRP-goat anti-mouse IgG secondary antibodies were diluted 1:3000 in 0.5% *w*/*v* skim milk solution and added to the plates. Plates were incubated to bind the secondary antibodies, then they were washed. Finally, OPD substrate was added (100 µL/well) and left to react for 20 min, then the stop solution (1N sulphuric acid) was added (100 µL/well), and plates were measured for absorbance using a plate reader.

### 2.8. Opsonization Assay

An opsonization assay was employed to establish the bactericidal inhibition of vaccine antisera, as reported previously [6,7] using ‘10-fold diluted sera’ to detect more significant opsonic antisera and thus the most promising antigens for future vaccine development. Two clinical GAS isolates were used: D3840 (nasopharynx swabs) and ACM-2727 (Royal Brisbane Hospital). These strains have highly conserved p145/J8i in their M-protein sequence. In addition, another two strains, NS1944 and NS5582, which have mutant J8i sequences, are only 75% and 58.3% conserved compared to the original J8i sequence, respectively, where mutations are located at the *C*-terminus of J8i (Figure 1). Briefly, high-quality antibodies recognize and bind to GAS and induce antibody-dependent phagocytosis of bacteria by macrophages. For each GAS strain, bacteria were cultured by transferring a single colony to Todd–Hewitt broth with 5% yeast extracts in a 50 mL Falcon tube and incubating at 37 °C for 24 h to obtain a concentration of 10^7^ CFU/mL. The culture is then diluted 100-fold in PBS. In a 96-well microplate, a 10 µL volume of the diluted culture was transferred, followed by adding 10 µL of heat-inactivated (50 °C, 10 min) murine sera and 80 μL of horse blood, the contents were mixed, and the microplates were incubated. The opsonization activity was calculated as the percentage reduction in CFU compared to the PBS negative control group sera. The assay was performed in duplicate. The bactericidal/inhibitory effect against the GAS strains was presented as a percentage compared to the non-inhibited control (Equation (1)). In addition, cross-clade inhibition can be calculated using variance of inhibition % among the four tested strains, and opsonization efficacy represented as log reduction in GAS bacteria can be normalized by the titres to produce an opsonic capacity index to rank the tested peptide vaccines in their potential opsonic capacity, regardless of their current immunogenicity in the current experiment (Equation (2)). The seeded count of GAS bacteria per test replicate is 10^3^ CFU, and the percentage inhibition can be converted to log reduction first before normalization by serum anti-peptide antibody titres (Equation (2)).

(1) $$Inhibition\;\%=[\;1-\frac{{CFU}_{Sera}}{{CFU}_{Medium}}]\times 100$$

(2) $$Opsonic\;Capacity\;Index=\frac{{Log}_{10}1000-{Log}_{10}[10\times \left(100-inh\%\right)]}{{Log}_{10}anti-peptide\;IgG\;titers}$$

### 2.9. Data Analysis

The main statistical test employed was one-way ANOVA, followed by Dunnett’s multiple comparisons test. Significance was set to *p* < 0.05 (*), *p* < 0.01 (**), *p* < 0.001 (***), and *p* < 0.0001 (****). In addition, OriginPro 2020 (OriginLab Corporation, Northampton, MA, USA) and GraphPad Prism v8 (GraphPad Software Inc., San Diego, CA, USA) were used for the mathematical and statistical presentation of the data.

## 3. Results

### 3.1. Peptide Synthesis and Characterization

**Figure 4:**
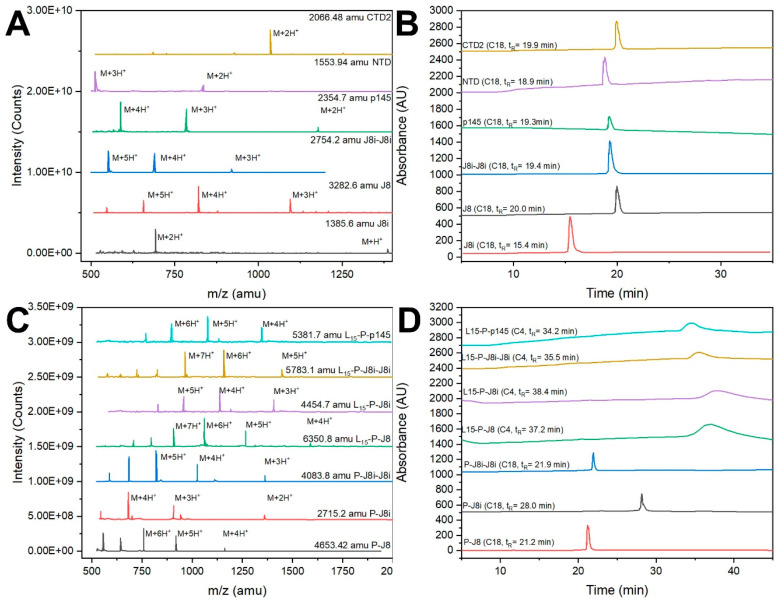
Synthesized peptides: (**A**,**B**) ESI-MS spectra and HPLC chromatograms of B-cell antigens, (**C**,**D**) ESI-MS spectra and HPLC chromatograms of T-helper epitope conjugates with B-cell antigens, with or without 15-mer polyleucine.

Peptides were synthesized using Fmoc-based SPPS (Figure 4, Table 1) and their purity and identity were confirmed using RP-HPLC and ESI-MS, respectively. The synthesized peptides included four self-adjuvanted polyleucine-based vaccines (L_15_-P-J8, L_15_-P-J8i, L_15_-P-p145, and L_15_-P-J8i-J8i). In addition, the synthesized peptides also included antigens either conjugated to PADRE T-helper epitope (P-J8i, P-J8, P-J8i-J8i, P-p145) or those physically mixed with it (P+J8i, P+J8, P+J8i-J8i, P+p145, P+NTD, P+CTD2).

### 3.2. Size and Shape of Self-Assembled Nanovaccines

**Figure 5:**
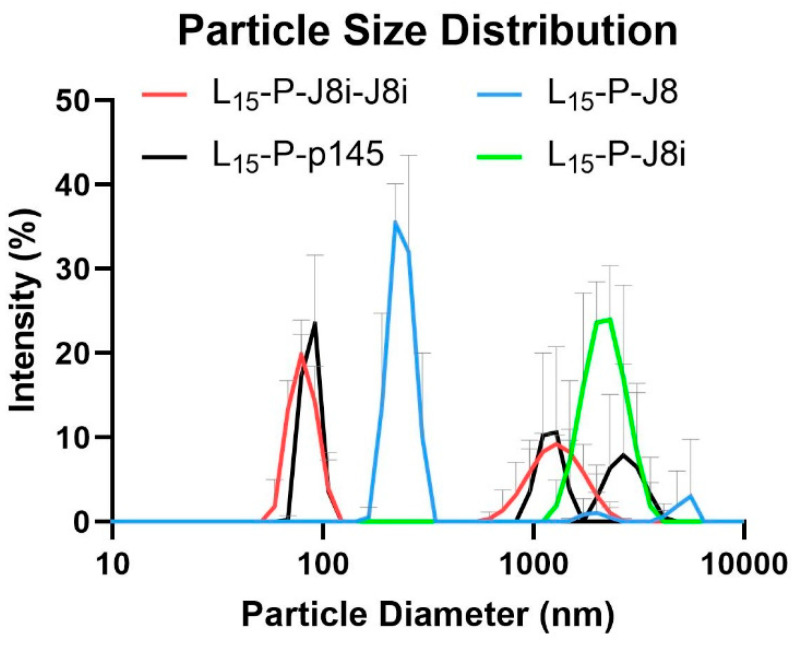
Size distribution of self-assembled 15-mer polyleucine vaccines in PBS via DLS.

**Figure 6:**
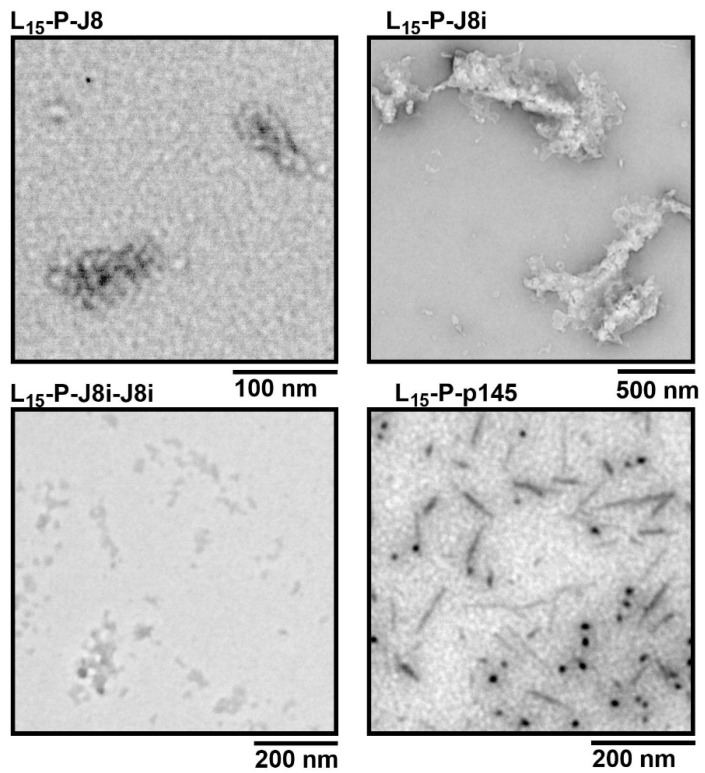
TEM electromicrographs of self-assembled 15-mer polyleucine vaccines in PBS.

Peptides were dissolved in PBS, and the size distribution of self-assembled polyleucine peptide nanovaccines was evaluated via DLS. L_15_-P-J8i self-assembled into microparticles with a peak diameter of 2.2 ± 0.3 µm, including aggregates at a larger size, as the polydispersity index was very high, 1.0, and this size was significantly larger than the other polyleucine peptide vaccines. This was attributed to the small size of the hydrophilic J8i peptide compared to the hydrophobic sequence of PADRE and L_15_ at the construct’s *N*-terminus (Figure 5). L_15_-P-J8, L_15_-P-p145, and L_15_-P-J8i-J8i self-assembled mainly into nanovaccines with peak sizes of 235 ± 13 nm, 87 ± 4 nm, and 81 ± 4 nm, with polydispersity indices of 0.62 ± 0.08, 0.87 ± 0.14, and 0.73 ± 0.05. L_15_-P-p145, and L_15_-P-J8i-J8i also distinctly included aggregates at microparticles of 1.8 ± 0.8 µm and 1.3 ± 0.2 µm in diameter. L_15_-P-J8, L_15_-P-p145, L_15_-P-J8i, and L_15_-P-J8i-J8i self-assembled into micro-/nanoparticles with z-average values of 484 ± 38, 427 ± 158, 2818 ± 650, and 135 ± 3 nm, respectively. Moreover, the TEM micrographs showed sizes that matched the DLS measurements (Figure 6).

### 3.3. Secondary Structural Evaluation of Peptide Antigens and Vaccines

**Figure 7:**
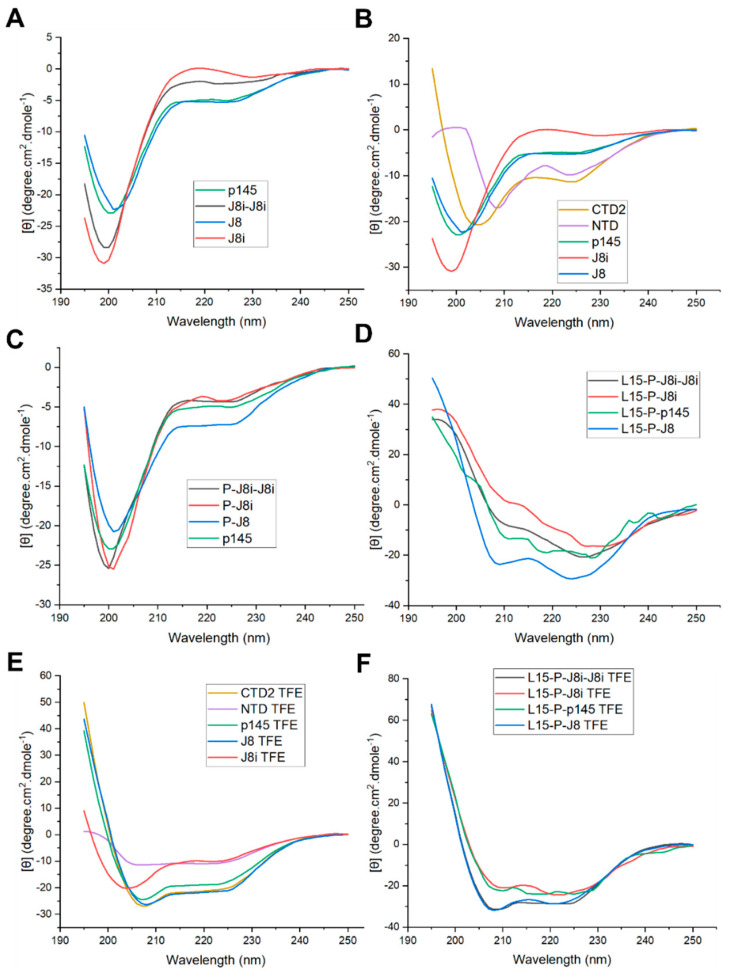
Circular dichroism measurements of the peptide vaccines and antigens, including (**A**) new and reported B-cell antigens, (**B**) J8-derivatives of B-cell antigens, (**C**) T-helper epitope conjugates to B-cell antigens, (**D**) polyleucine-adjuvanted vaccines, and (**E**,**F**) peptide antigens and vaccines in PBS including trifluoroethanol.

The secondary structures of the synthesized peptides were evaluated using CD spectropolarography (Figure 7 and Table S1). J8-derivative B-cell antigens (J8, J8i, J8i-J8i, and p145) were mainly random coil (73 to 87%) with some helical structures (25 to 13%). The random coil or conformation-less content followed the length of the B-cell antigens, where the minimalist peptide J8i had only a 13% α-helix content, compared to p145, J8i-J8i, and J8, which had 17.25, 25%, and 27%, respectively. The conjugation of PADRE universal T helper also increased the helical content of the whole construct, where P-J8, P-J8i, and P-J8i-J8i had 31%, 25%, and 23% helical contents, respectively.

Recently, it has been reported that peptides adopt a native conformation in the vicinity of lipid membranes in vivo. Researchers attempted to mimic such an effect on peptide secondary structures by including small unilamellar liposomes or by including a cosolvent such as TFE. The secondary structures were evaluated in the presence of TFE for comparison [32,33]. The inclusion of TFE increased helical contents of the epitopes, where J8i, p145, J8i-J8i, and J8 had 41%, 67%, 71%, and 74%, respectively. Moreover, the conjugation to PADRE rendered P-J8, P-J8i, and P-J8i-J8i peptides of equivalent and high helical contents, ranging from 74 to 77%, in the presence of TFE. CTD2 and NTD had relatively high α-helical contents of 45% and 55% in PBS, which increased to 55% and 65% in TFE/PBS, respectively. Polyleucine vaccine constructs had relatively high helical contents >75% in PBS and increased to completely α-helical (>92%) in TFE/PBS.

These peptide antigens were adjuvanted with complete Freund’s adjuvant (CFA) and investigated for their immunogenicity, quality of generated antibodies, and antisera’s opsonization efficacy, following single immunization of C57BL/6 mice or three immunizations for polyleucine immunized C57BL/6 mice, followed by immunological analyses of the murine sera. This aimed to investigate the impacts of altering the B-cell epitope (J8-derivatives), the effect of T-helper conjugation on the B-cell epitope, and employing polyleucine as an adjuvant on the immunogenicity, quality, and efficacy of generated immune responses in the presence of another infection, i.e., *S. aureus*.

### 3.4. Immunological Evaluation of Mouse Sera

**Figure 8:**
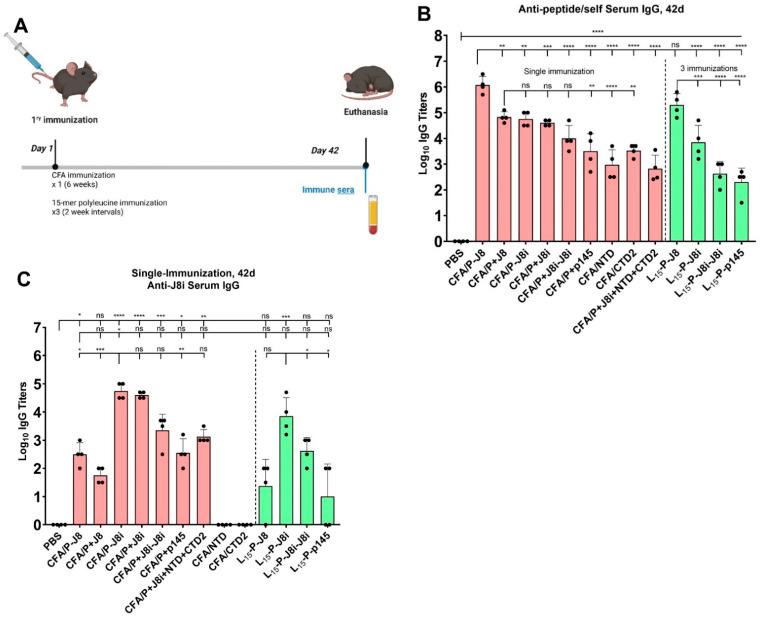
Immunization of C57BL/6 mice and immunological analyses of sera: (**A**) immunization schedule, (**B**) serum anti-peptide IgG titres, each against the intended peptide vaccine antigen, and (**C**) serum anti-J8i using ELISA. One-way ANOVA with Dunnett’s multiple comparisons test was used for statistics. Significance was set to *p* < 0.05 (*), *p* < 0.01 (**), *p* < 0.001 (***), and *p* < 0.0001 (****).

Initially, due to the existing postnatal infection of the C57BL/6 mice with *S. aureus* (Figure S1), serum IgG titres against GAS bacteria were detected; however, no serum IgG titres were detected against the peptide antigens (Figure 8 and Figure S2). The mice were immunized with each peptide vaccine (n = 4), and sera were collected and analysed for immune responses after 42 days post-immunization, against various coating antigens J8, J8i, p145, NTD, and CTD2, and whole inactivated GAS (Figure 8A). Anti-peptide ELISAs were performed among the different mouse groups, where each mouse group’s sera were evaluated against the peptide antigen with which it was immunized. This represented an evaluation and means of comparison among the antigens’ immunogenicities.

The CFA/P-J8 immunized mouse group was the most immunogenic in generating anti-peptide serum IgG titres (Figure 8B), compared to the other groups, followed by CFA/P+J8, CFA/P-J8i, CFA/P+J8i-J8i, and CFA/P+J8i; these vaccines generated lower serum anti-peptide IgG titres (*p* < 0.01) than CFA/P-J8 but were similar to each other (*p* > 0.05). This demonstrated the superior immunogenicity of J8 compared to the other antigens, and improved immunogenicity due to the conjugation of the helper T-cell epitope, as CFA/P+J8-immunized group sera had lower anti-J8 IgG titres than those in the sera of the CFA/P-J8-immunized group (*p* < 0.01). The newly tested peptides CFA/P+NTD and CFA/P+CTD2 generated fewer serum anti-peptide IgG titres (log_10_ IgG titres of 3 to 3.5) compared to the CFA/P+J8- and CFA/P+J8i-immunized groups (Log_10_ anti-peptide IgG titres of 4.5 to 5), suggesting that NTD and CTD2 are less immunogenic than J8 or J8i. The rank order of absolute immunogenicity was as follows: P-J8 > P+J8 ≈ P-J8i ≈ P+J8i ≥ P+J8i-J8i > P+p145 ≈ CTD2 ≈ NTD, with either adjuvant polyleucine or CFA.

Anti-J8i ELISA was conducted on immunized mouse sera. As J8i is the native minimalist peptide sourced from p145 within the GAS bacterium M-protein sequence, it was expected that anti-J8i titres would be highly relevant to the immunogenicity of J8i-based vaccines (Figure 8C). Although it is a completely native peptide derived from M-protein, its short minimalist sequence may prevent it from adopting its native conformation. Anti-J8i serum IgG titres were highest for CFA/P-J8i-immunized mouse group sera, similar to those generated by CFA/P+J8i-immunized mice (*p* > 0.05). The CFA/P+J8i-J8i- and L_15_-P-J8i-J8i-immunized mouse groups had slightly lower anti-J8i IgG titres in mouse sera (*p* > 0.05 and *p* < 0.05, respectively) compared to CFA/P-J8i. Meanwhile, p145 vaccines (CFA/P+p145 and L_15_-P-p145) generated significantly lower anti-J8i IgG titres in the sera (*p* < 0.01 and *p* < 0.05) compared to CFA/P-J8i and L_15_-P-J8i, respectively. In sum, the anti-J8i serum IgG titres generally increased with the usage of more minimalistic antigens (in the rank order of P-J8i ≈ P+J8i > P+J8i-J8i > P+p145 > J8), and most of the generated anti-J8i IgG did not recognize J8 but recognized p145 to some extent. The Shapiro–Wilk test was conducted, at α = 0.05, to assess the normality of variances for the anti-J8i IgG immunological responses of the mouse groups, and they were normally distributed except for anti-J8i IgG for the CFA/P+J8i-J8i and L_15_-P-p145 vaccines.

**Figure 9:**
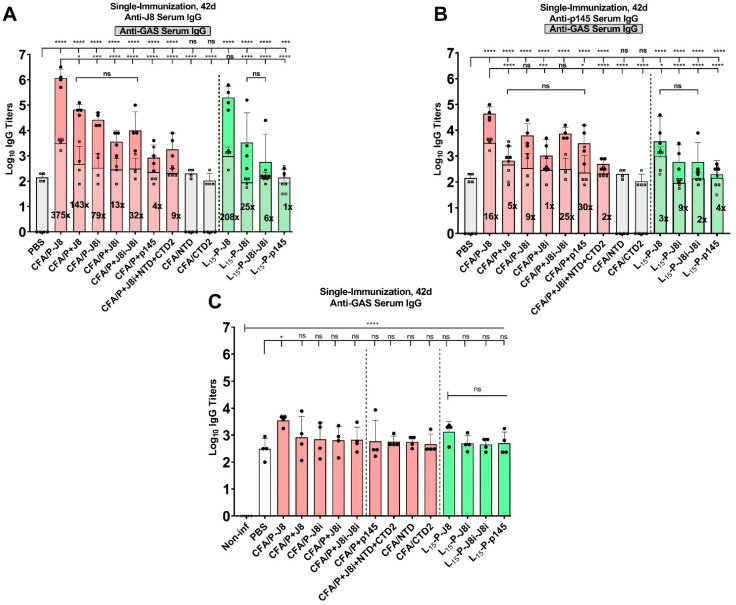
Immunological analyses of C57BL/6 mice sera. (**A**) Serum anti-J8 and anti-GAS IgG titres, where shaded bars represent the anti-GAS titres, determined using the ELISA. The numerical factor at the base of each column represents the value of the gap between anti-J8 and anti-GAS IgG titres. (**B**) Serum anti-p145 and anti-GAS IgG titres, where shaded bars represent the anti-GAS titres, determined using ELISA. The numerical factor at the base of each column represents the value of the gap between anti-p145 and anti-GAS IgG titres, and (**C**) serum anti-GAS using the ELISA. One-way ANOVA with Dunnett’s multiple comparisons test was used for statistics. Significance was set to *p* < 0.05 (*), *p* < 0.001 (***), and *p* < 0.0001 (****).

Anti-J8 ELISAs were performed among the different mouse groups compared to PBS and to CFA/P-J8 as negative and positive control groups, respectively (Figure 9A). The CFA/P+J8-immunized mouse group was slightly less immunogenic compared to CFA/P-J8, *p* < 0.05. Three immunizations with L_15_-P-J8 triggered equivalent immune responses to CFA/P-J8, *p* > 0.05, and all other mouse groups were significantly less immunogenic compared to CFA/P-J8. Furthermore, CFA/P-J8i was significantly less immunogenic than CFA/P-J8 (*p* < 0.001 and *p* < 0.0001, respectively). CFA/P+J8i was significantly less immunogenic than CFA/P+J8 (*p* < 0.05). In addition, CFA/P+J8i-J8i-immunized mouse groups had significantly lower anti-J8 serum IgG titres compared to CFA/P-J8 (*p* < 0.0001) but were similar to those generated by the CFA/P+J8-immunized mouse group (*p* > 0.05), whereas CFA/P+p145 generated significantly lower anti-J8 serum IgG titres compared to CFA/P-J8 or CFA/P-J8i (*p* < 0.0001 and *p* < 0.01, respectively). Finally, L_15_-P-J8i and L_15_-P-J8i-J8i generated similar serum anti-J8 IgG titres to one another (*p* > 0.05), while L_15_-P-p145 was significantly less immunogenic (*p* < 0.001). In sum, the generation of anti-J8 IgG titres in the serum was in the following rank order with either adjuvant system: P-J8 > P+J8 ≈ P-J8i > P+J8i-J8i > P+J8i >> P+p145. Further, the PBS group of infected mice, as well as CFA/NTD and CFA/CTD2 immunized mice, did not generate detectable anti-J8, which shows J8-specific responses and suggests that the peptide is not shared with *S. aureus* bacterial surface antigens. The Shapiro–Wilk test was conducted, at α = 0.05, to assess the normality of variances for the anti-J8 IgG immunological responses of the mouse groups, and they were normally distributed except for anti-J8 for the L_15_-P-J8i-J8i vaccine.

Anti-p145 ELISA was conducted on immunized mouse sera. As p145 is the native epitope source (of J8 and J8i) in the GAS M-protein, it was expected that anti-p145 titres would be highly relevant to antibody quality or the avidity of the generated immune responses (Figure 9B). Anti-p145 serum IgG titres were the highest for CFA/P-J8 immunized mouse group sera, potentially on account of the immunogenicity of the P-J8 peptide. The CFA/P+p145- and L_15_-P-p145-immunized mouse groups had slightly lower anti-p145 IgG titres in mouse sera (*p* < 0.05) compared to CFA/P-J8. Meanwhile, CFA/P+J8, CFA/P-J8i, CFA/P+J8i, and CFA/P+p145 generated similar serum titres of anti-p145 IgG (*p* > 0.05). In addition, the L_15_-P-J8-, L_15_-P-J8i-, and L_15_-P-J8i-J8i-immunized mouse groups also had similar serum anti-p145 IgG titres (*p* > 0.05). Surprisingly, when comparing CFA/P-J8- and CFA/P+J8-immunized group sera, a dramatic reduction in anti-p145 was observed when PADRE was not conjugated (*p* < 0.0001). The Shapiro–Wilk test was conducted, at α = 0.05, to assess the normality of variances for the anti-p145 IgG immunological responses of the mouse groups, and they were normally distributed except for anti-p145 IgG for the L_15_-P-J8i-J8i vaccine.

An Anti-GAS ELISA was conducted on mouse immune sera (Figure 9C). CFA/P-J8 generated the highest serum anti-GAS IgG titres. Nonetheless, L_15_-P-J8 generated anti-GAS serum IgG titres similar to those generated by CFA/P-J8. However, all the other groups, including the CFA/P+J8-immunized mouse group, generated lower anti-GAS titre levels compared to CFA/P-J8 (*p* < 0.05). Polyleucine-adjuvanted mouse groups generated similar anti-GAS IgG titres to each other, and L_15_-P-J8 generated similar anti-GAS IgG titres to the CFA/P-J8 immunized mouse group, after three immunizations. However, L_15_-P-J8i, L_15_-P-J8i-J8i, and L_15_-P-p145 generated lower but non-significantly different anti-GAS serum IgG compared to CFA/P-J8 or L_15_-P-J8 (*p* > 0.05). In addition, the NTD- and CTD2-immunized mouse groups generated significant but modest anti-GAS titres, which suggests that the epitopes are not as immunogenic as J8 (or its derivatives) but represent a promising approach for developing a multivalent vaccine against GAS. It was also observed that the PBS group of infected mice generated some anti-GAS IgG titres (log_10_ serum IgG of 2.0 to 3.0) compared to naïve sera from the non-infected PBS group (Figure S2). This suggests that there are some shared epitopes between surface antigens of GAS and *S. aureus* bacteria. The Shapiro–Wilk test was conducted, at α = 0.05, to assess the normality of variances for the anti-GAS IgG immunological responses of the mouse groups, and they were normally distributed except for anti-GAS IgG for the CFA/P+J8i and CFA/P+J8i-J8i vaccines.

### 3.5. Opsonization Efficacy Evaluation of Mouse Sera

Sera of the immunized mouse groups were evaluated for their opsonic bactericidal inhibition activity against four GAS clinically isolated strains, two of which were standard strains with a conserved p145/J8i sequence (D3840 and ACM2727), while the other two clinical strains were mutant virulent strains with mutated p145/J8i sequences (NS1944 and NS5582) (Figure 1). Pre-immunization sera did not significantly impact the opsonization efficacy of any of the tested strains, *p* > 0.05 (Figure S3).

**Figure 10:**
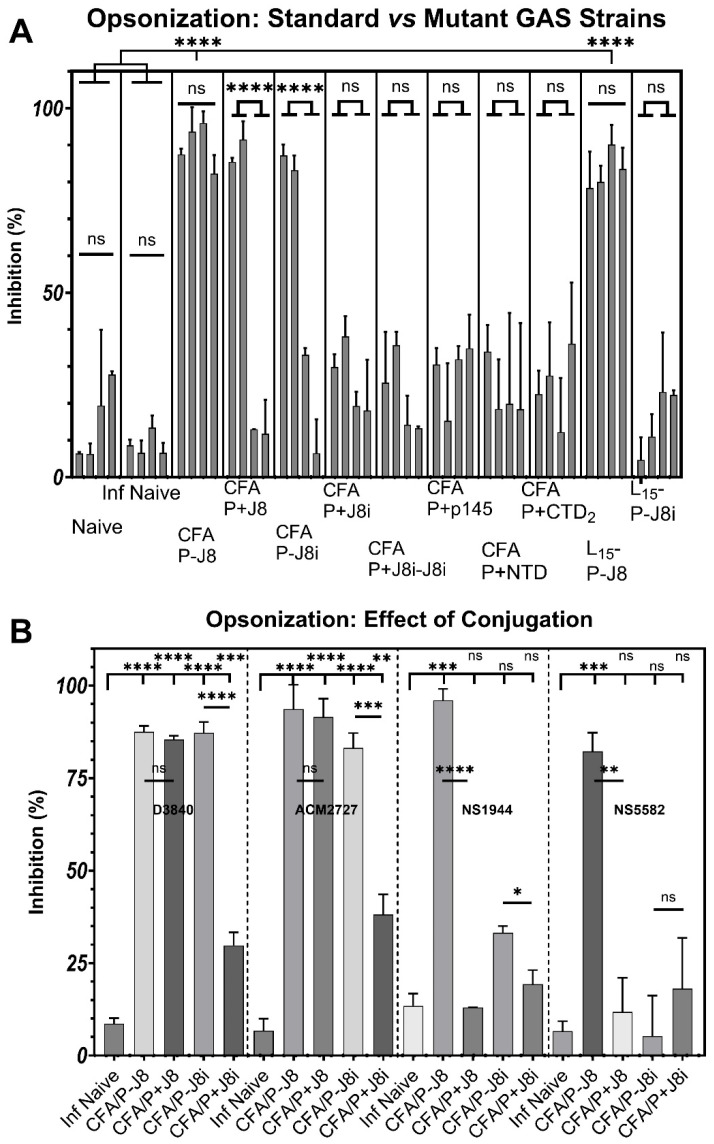
GAS opsonization inhibition via (**A**) antisera of immunized mouse groups against four clinical strains—two standard strains (D3840 and ACM2727) and two mutant virulent strains (NS1944 and NS5582)—where each bar for a given vaccine represents bactericidal inhibition against a specific GAS strain from left to right in the order of D3840, ACM2727, NS1944, and NS5582, respectively. (**B**) The influence of B-cell antigen conjugation to the T helper on GAS opsonization inhibition of immunized mouse groups against four clinical strains—two standard strains (D3840 and ACM2727) and two mutant virulent strains (NS1944 and NS5582). Statistics were compared for bactericidal inhibition between the two standard and the two mutant strains and compared to the PBS negative control group. One-way ANOVA with Dunnett’s multiple comparisons test was used for statistics. Significance was set to *p* < 0.05 (*), *p* < 0.01 (**), *p* < 0.001 (***), and *p* < 0.0001 (****).

**Figure 11:**
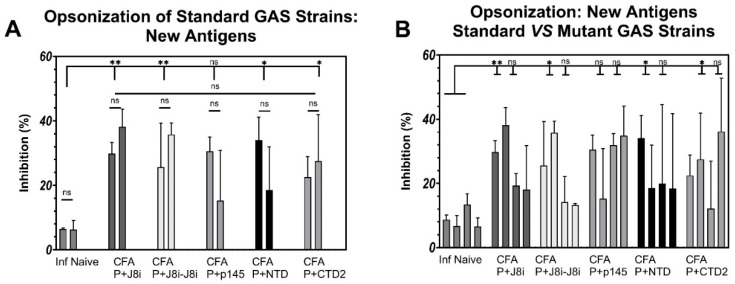
GAS opsonization inhibition of immunized mouse groups, comparing vaccine groups with new antigens and J8-derivative antigens against (**A**) the standard two GAS strains or (**B**) four standard and mutant GAS strains, respectively. Statistics compare bactericidal inhibition across all tested clinical GAS strains between standard and mutant strains and between immunized groups and the PBS negative control group. One-way ANOVA with Dunnett’s multiple comparisons test was used for statistical analysis. Significance was set to *p* < 0.05 (*), and *p* < 0.01 (**).

The standard strains were effectively opsonized by many antisera, >80%, against many vaccine antigens, including CFA/P-J8, CFA/P+J8, CFA/P-J8i, and L_15_-P-J8, compared to PBS control group sera (*p* < 0.0001) (Figure 10A). The T-helper-conjugated vaccine CFA/P-J8i generated more bactericidal antisera against the standard GAS strains compared to antisera against CFA/P+J8i, *p* < 0.001 (Figure 10B). In addition, the antisera against L_15_-P-J8i vaccines did not exhibit opsonic efficacy against the standard GAS strains. In contrast, antisera against CFA/P+CTD2, CFA/P+NTD, CFA/P+J8i, and CFA/P+J8i-J8i significantly but modestly generated opsonic antisera against GAS standard strains (≤50%, *p* < 0.05, Figure 10A), with CFA/P+J8i-J8i and CFA/P+J8i vaccines generating more significant opsonic antibodies (*p* < 0.01) compared to PBS control group sera. In contrast, the performance of the antisera generated by the vaccines was very different against mutant GAS strains. The two mutant strains NS1944 and NS5582 resisted opsonization by the antisera generated by the vaccines with many of the tested antigens: CFA/P+J8, CFA/P+J8i, CFA/P-J8i, CFA/P+J8i-J8i, CFA/P+p145, CFA/P+NTD, and CFA/P+CTD2 (*p* > 0.05, Figure 10 and Figure 11). Only antisera against P-J8 vaccines, adjuvanted with either CFA or 15-mer polyleucine, elicited effective opsonic bactericidal activity against all tested GAS strains, including the mutant virulent GAS strains (*p* < 0.001, Figure 10 and Figure 11).

The new B-cell antigens NTD and CTD2 (CFA/P+NTD and CFA/P+CTD2) generated antisera that were moderately opsonic against the standard GAS strains (*p* < 0.05 Figure 11A); however, they had no significant opsonic activity against the mutant strains, despite being conserved in their M-protein sequences (*p* > 0.05, Figure 1 and Figure 11B). Notably, the J8i and J8i-J8i antigens’ (CFA/P+J8i and CFA/P+J8i-J8i) antisera were more opsonically effective (*p* < 0.01) against standard GAS strains compared to NTD and CTD2 (*p* < 0.05) when either was compared to the PBS negative control group, but they were all more or less at similar modest inhibition levels ranging between 20% and 40% (Figure 10A and Figure 11A).

## 4. Discussion

### 4.1. Size and Secondary Structural Characterization of Synthesized Peptide Constructs

Peptide antigens were successfully synthesized. The 15-mer polyleucine self-assembled peptide vaccines were characterized for their size distribution using DLS and TEM. The most stable nanovaccine size, with a smaller aggregation extent, was observed for L_15_-P-J8, and this was attributed to the hydrophilicity of J8 B-cell antigen, which represented a significant portion of the sequence, compared to J8i-J8i or p145. Therefore, the hydrophilicity of B-cell antigens at *C*-termini played a major role in the self-assembled size of the peptide constructs, where a longer hydrophilic sequence resulted in smaller, more monodisperse, and less aggregating nanovaccines; the rank order of peptides’ hydrophilicity was J8 > J8i-J8i > p145 > J8i.

The peptide constructs were dissolved in PBS and characterized for their secondary structural conformation using CD spectroscopy, both in the presence and absence of TFE co-solvent. It was observed that the helical content increased with the length of the B-cell antigen employed. The helical content demonstrated that J8 was precisely designed to match the p145 native peptide structure, after omitting the autoimmune sequence at its *N*-terminus. In addition, this also shows that J8i-J8i dimer and J8i minimalist peptides lack the necessary residues to convey a matching native helical structure to that observed for the p145 peptide. In addition, the conjugation of PADRE T helper to B-cell antigens increased the helical conformation content of the constructs. The impact of PADRE on the short J8i sequence was higher in the induction of helicity compared to the J8i-J8i dimer, probably due to the greater proportion of the short epitope being influenced by neighbouring residues to PADRE. The addition of TFE to peptide solutions increased the helical conformation contents for many peptides; however, the helical content increase in J8i peptide was modest compared to the increase observed for the other B-cell antigens J8, J8i-J8i, and p145. This demonstrated that J8i still lacked the necessary residues to adopt a native helical conformation; however, p145, J8i-J8i dimer, and J8 epitopes were of equivalent helical contents ranging from 67 to 74%. Further, the conjugation of PADRE increased the helical content of J8i, in the presence of TFE; however, it did not significantly increase J8 or J8i-J8i dimer, compared to constructs without PADRE, in the presence of TFE. This demonstrated that conjugation of PADRE not only helps with generating robust, long-lasting immune responses as a universal helper T-cell epitope for C57BL/6 mice [6,7] but also assists in adopting a native structural conformation for short neighbouring epitopes like J8i. However, it remains to be tested whether the peptide structure influenced their immunogenicity and the quality of triggered responses in the mice.

### 4.2. The Immunological Evaluation of Mouse Sera: Immunogenicity and Immune Response Quality

The peptide vaccines were prepared and used to immunize female C57BL/6 mice. Murine sera were collected and analysed for immunological responses using ELISA against various antigens, including the immunizing peptides themselves (Figure 8B), J8, J8i, and p145, as well as the inactivated GAS bacteria. ELISA against the immunizing peptide antigens provided the highest lgo_10_ titre responses, thus supporting that they are most suited to evaluating vaccine immunogenicity. In terms of absolute immunogenicity (anti-peptide IgG titres), conjugation to PADRE T helper enhanced the immunogenicity over a physical mixture between PADRE and B-cell antigen groups. In addition, the immunogenicity of J8-derivative vaccine J8 was the greatest, followed by J8i, J8i-J8i, and then p145.

ELISA against J8i peptide demonstrated higher responses for J8i-based vaccines over those employing J8 or p145 antigens (Figure 8B). However, there are gaps of 0.9 and 1.6 log_10_ units between anti-J8i and anti-p145 IgG titres of CFA/P-J8i and CFA/P+J8i, respectively. This suggests that the immunogenicity of J8i is higher than p145, close in ELISA unit values to anti-J8 IgG titres for the CFA/P-J8-immunized group; however, the high-quality antibodies generated by J8i-vaccines that recognized p145 were only 12.5% or 2.5%, for PADRE conjugated to, or physically mixed with, J8i vaccine antigens, respectively. This also demonstrated that the conjugation of PADRE to J8i improved the quality of generated antibodies rather than immunogenicity, potentially by supporting a more native helical structure for J8i. In sum, the anti-J8i serum IgG titres generally increased with the usage of more minimalistic antigens, and most of the generated anti-J8i IgG did not recognize J8 but recognized p145 more slightly. Further, the conjugation of PADRE to short J8i improved the quality of generated anti-J8i serum IgG antibodies in terms of recognition of p145 antigen, compared to a CFA/P+J8i physical mixture.

The ELISA against the J8 peptide demonstrated higher responses for CFA/P-J8 and CFA/P-J8i vaccines over CFA/P+J8 and CFA/P+J8i, respectively (Figure 9A). This demonstrated the importance of conjugating the PADRE T-helper epitope to short peptide antigens for enhancement of their native structure and for delivery to the same lymph node, which was often reported to enhance immunogenicity, despite the presence of freely mixed PADRE T-helper epitope. Although CFA contains inactivated mycobacteria, which form a source of numerous helper T-cell epitopes, and thus the enhanced immunogenicity could be attributed to the increased adoption of a helical secondary structure of the B-cell epitope induced by the conjugated T helper. In addition, serum anti-J8 IgG titres generated by the vaccines were highest for CFA/P+J8, followed by P+J8i-J8i, P+J8i, and then P+p145. Surprisingly, the gap value between anti-GAS titres and anti-J8 titres was smaller for mixed non-conjugated T-helper-based vaccines, like CFA/P+J8 and CFA/P+J8i, compared to their conjugated counterparts, CFA P-J8 and CFA/P-J8i, by 3- to 5-fold, and the gap was wider with shorter B-cell antigen sequences (Figure 9A). This suggests that recognition of GAS bacteria might be slightly better with the physical mixture, thus producing higher-quality antibodies; however, due to the interference of *S. aureus* infection in anti-GAS titres, it is difficult to provide definitive conclusions without the opsonization assay outcomes.

The ELISA against the p145 peptide demonstrated the highest responses for the CFA/P-J8 vaccine (Figure 9B). The anti-p145 serum IgG titres generally increased with the increase in peptide immunogenicity; however, the conjugation of PADRE to short J8i or using the dimer J8i-J8i, i.e., CFA/P-J8i- and CFA/P+J8i+J8i-immunized mouse groups, improved the magnitude of triggered immune responses compared to P+J8i or P+p145. Further, the conjugation of PADRE to J8 dramatically improved its anti-p145 titres, despite the fact that J8 is relatively long and equipped with GCN-4 at both termini. In addition, the gaps between anti-p145 titres and anti-GAS titres (Figure 9C) were noticeably smaller than any other ELISA peptide-antigen coat; the gaps varied between 2- and 30-fold for all p145-based vaccine derivatives and only varied 1- to 16-fold for J8- and J8i-based vaccines, which suggests that p145 can be employed for ELISA antigen coating as a measure of antibody quality or immune response avidity.

Anti-GAS IgG titres often correlate best with opsonization and represent the overall quality of generated antibodies and their ability to recognize GAS (Figure 9C). The anti-GAS IgG serum titres were similar among various antigens; however, the conjugation of PADRE was essential to enhance the anti-GAS titres of the J8 and J8i peptides. In addition, there was an observed interference by the existing *S. aureus* infection. Thus, solutions to this issue include either blank subtraction (pre-immunization from post-immunization sera) or the usage of alternative distinctive (non-shared) peptides or proteins from GAS as ELISA coating antigens, to restore the specificity of the assay. Alternatively, NTD and CTD generated significant but very low serum anti-GAS IgG titres; therefore, they should be subjected to further improvements in future research.

### 4.3. In Vitro Opsonization Efficacy of Mouse Sera Against Standard and Mutant GAS Strains

The murine antisera against vaccines were evaluated for their opsonic bactericidal efficacy in vitro against two standard and two mutant GAS strains. The conjugation of PADRE to J8 was not necessary to generate effective opsonic antibodies against the standard GAS strains, as CFA/P+J8 was effective in generating opsonic antibodies against standard GAS strains (Figure 10B). In contrast, the conjugation of PADRE to J8i was necessary to generate opsonic antibodies against standard GAS strains, as CFA/P+J8i did not generate effective opsonic antibodies compared to CFA/P-J8i, thus PADRE conjugation at the *N*-terminus and CFA lipids potentially contributed to restoring the native conformation of the short J8i epitope. Surprisingly, L_15_-P-J8i was also ineffective in generating opsonic antibodies against the standard GAS strains, which suggests that the β-like protein conformation structure induced by the polyleucine at one end, and random coil residues at the *C*-terminus of the short J8i peptide at the other end, may have obscured the structure of the short J8i sequence.

The opsonic efficacy of immunized mice antisera was very different from that of the mutant GAS strains. Only L_15_-P-J8 and CFA/P-J8 generated highly opsonic antisera against mutant GAS strains, compared to the antisera against other vaccines, including CFA/P+J8. In the context of generating opsonic antibodies using J8-derivative antigens against the mutant virulent GAS strains, it is apparent that not only is J8 antigen, including GCN-4 sequences, necessary for the opsonic activity but also its conjugation at the B-cell antigen’s *N*-terminus to the PADRE T-helper epitope, which aids in helical conformation induction and antigen presentation. Thus, these modifications to the vaccine antigen supported both the vaccine antigen’s immunogenicity and efficacy, especially against mutant GAS strains; the same effect could hold for T-helper carrier protein sources like diphtheria toxins. Since the mutations in NS1944 and NS5582 in p145/J8i sequences are located at its *C*-terminus (Figure 1), this suggests that the conjugation of PADRE and GCN-4 sequences to J8 was necessary for effective antigen presentation of the conserved *N*-terminal residues of J8i (Figure 10 and Figure 11), which could be the main source that generated opsonic antibodies against these mutant GAS strains. Meanwhile, conjugation of PADRE and use of lipid-based adjuvants were adequate to generate effective opsonic antibodies against ‘standard GAS strains’ where *C*-terminus residues of p145/J8i remain conserved in their M-protein sequences (Figure 1). In contrast, antisera against CFA/P+NTD and CFA/P+CTD2 were only moderately opsonic against standard GAS strains and were not effective against the mutant GAS strains (Figure 10), despite the fact that their sequences are conserved in both GAS strain types, potentially due to their modest immunogenicity. Thus, further development is required for these new antigens to boost their immunogenicity and the quality of the generated antibodies to be as effective as J8; among such modifications are conjugation to PADRE, flanking with helical conformation-inducing sequences, or using a carrier T-helper source protein. These modifications could help promote the use of these new antigens in future vaccines against GAS infection. However, the new antigens NTD and CTD2 proved their usefulness as GAS-specific antigens in the presence of *S. aureus* infection, and thus could also be of diagnostic value.

**Figure 12:**
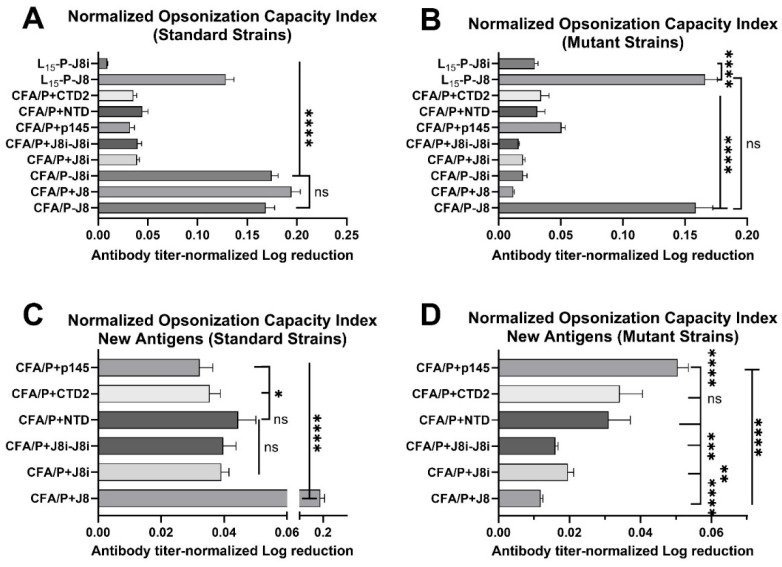
Opsonization capacity indices of antisera from immunized mouse groups (**A**,**C**), comparing vaccinated mouse groups and new antigens’ opsonization capacity indices against the two standard GAS strains, respectively. (**B**,**D**) Vaccinated mouse groups and new antigens’ opsonization capacity indices against the two mutant GAS strains, respectively. Statistics comparing the opsonization capacity indices among the antisera of the immunized groups against standard (left panels (**A**,**C**)) or mutant GAS strains (right panels (**B**,**D**)). One-way ANOVA with Dunnett’s multiple comparisons test was used for statistics. Significance was set to *p* < 0.05 (*), *p* < 0.01 (**), *p* < 0.001 (***), and *p* < 0.0001 (****).

The opsonic bactericidal inhibition of a given antisera is dependent on vaccine immunogenicity, i.e., the magnitude of generated antibody titres. Thus, normalization of the opsonic efficacy, or log reduction in seeded GAS bacteria, normalized by antigen-specific antibody titres was necessary to compare among the employed vaccine antigens (Figure 12), herein termed the opsonization capacity index. When comparing the opsonization capacity indices of the antisera against standard GAS strains (Figure 12A,C), it was observed that the antisera of CFA/P-J8-, CFA/P-J8i-, and CFA/P+J8-immunized groups were similar to each other, *p* > 0.05, and very significantly higher than all the other immunized groups, including L_15_-P-J8, *p* < 0.0001. This means that when the average generated anti-peptide antibody titres were taken into account, the antibodies of P-J8, P+J8, and P-J8i became equivalent in their opsonic activity against ‘the standard GAS strains’, which suggests that the role of PADRE conjugation to J8 supported the antigen’s immunogenicity and antigen presentation to immune cells rather than restoring the native structural conformation. However, the latter observation was noticeably more relevant for the J8i minimalist epitope (CFA/P-J8i), as it significantly reduced its antisera’s capacity index when it was not conjugated to PADRE (CFA/P+J8i). Overall, J8 antisera had the highest opsonization capacity index among physically mixed B-cell antigens (Figure 12C). However, when comparing opsonization capacity indices of antisera against the mutant strains, only antisera with J8 conjugated to PADRE T helper (CFA/P-J8 or L_15_-P-J8 vaccines) had very high and significant opsonic capacity indices compared to all other vaccines, *p* < 0.0001, and this opsonic capacity was comparable to that observed for the standard strains (Figure 12B). This is a very valuable finding because few-residue mutations, but not complete deletion, in J8i sequences did not prevent the generation of effective opsonic antibodies against these mutant GAS strains. Surprisingly, when opsonization was normalized by immunogenicity for ‘the physically mixed vaccines’, i.e., non-conjugated T-helper epitopes, and new antigen CFA/P+p145, CFA/P+CTD2, and CFA/P+NTD vaccines, their antisera had a significantly higher opsonization capacity, against the mutant strains, than J8, J8i, and J8i-J8i, *p* < 0.01, in the rank order of p145 >> CTD2 ≈ NTD >> J8i > J8i-J8i > J8 (Figure 12D). This suggests that these new B-cell antigens could be very promising vaccine antigens but require further development to boost their immunogenicity, thereby potentially achieving a similar potent opsonic efficacy to P-J8.

### 4.4. Relationships Among Immunological Responses and Opsonic Bactericidal Activity

**Figure 13:**
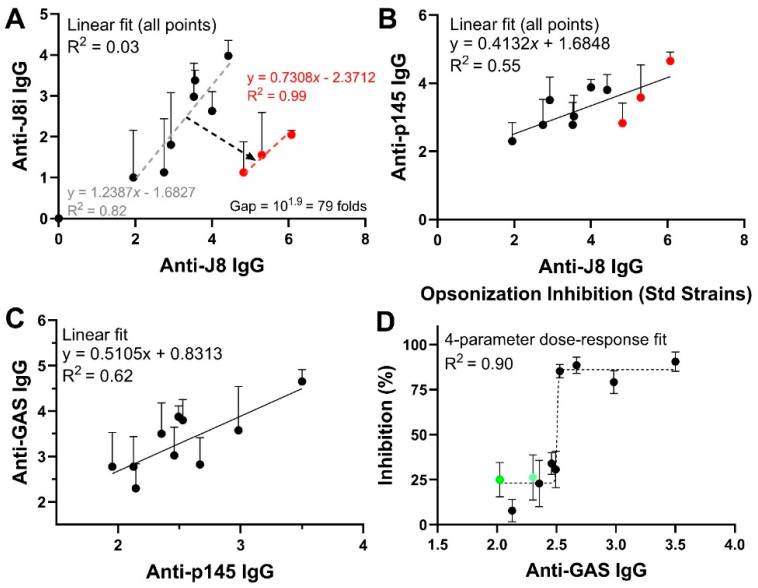
Correlations among the generated immune responses and opsonisation inhibition of standard GAS strains: between (**A**) serum anti-J8 and serum anti-J8i IgG titres (red circles represent J8-based vaccines datapoints), (**B**) serum anti-p145 and serum anti-J8 IgG titres (red circles represent J8-based vaccines datapoints), (**C**) serum anti-GAS and serum anti-p145 IgG titres, and (**D**) opsonization inhibition and serum anti-GAS IgG titres (green circles represent NTD and CTD2 datapoints).

The relationships among the immune responses were further investigated; anti-J8 serum IgG titres were plotted against anti-J8 IgG titres and against anti-p145 serum IgG titres (Figure 13A,B). A clear gap/x-offset (log_10_ of 1.9, ca. 80-fold, Figure 13A) was observed only for J8-based vaccine immunized groups between anti-J8i and anti-J8 IgG titres. The gap represented an 80-fold increase in anti-J8 IgG, for J8-based vaccines, at the expense of anti-J8i antibodies generated by the same vaccines, which could be due to antibodies generated against the non-native GCN-4 sequences. However, the gap/x-offset between J8 and J8i vaccines was almost completely closed when p145 was used as a coat for ELISA, i.e., anti-p145 serum IgG (Figure 13B), which demonstrates that ‘the extra anti-J8 IgG titres’ generated by J8-based vaccines, which caused the gap in Figure 13A, were recognized by p145, thus these are not irrelevant antibodies against the non-native GCN-4 sequence residues. This outcome suggests that p145 and J8 are more related to each other than J8i is related J8, and that the addition of GCN-4 sequences transformed the structure of J8i peptide, bring it closer in conformation to p145’s native structure within M-protein, and potentially restored the structure of unknown B-cell epitopes located within the J8i sequence. Thus, J8i potentially contains more than one B-cell epitope, whose structure was lost, especially when the minimalist J8i was used ‘unconjugated’ as a vaccine antigen. It remains to be seen whether further improvements over GCN-4 sequences can bring J8i even closer to p145, thereby closing the gap completely and enhancing the quality of generated antibodies, while boosting the antigen’s immunogenicity.

The relationships between efficacy and antigen-specific antibody titres were further investigated. The relationships between anti-GAS IgG titres and anti-p145 IgG titres and between anti-GAS IgG titres and opsonization efficacy against ‘the standard strains’ were explored (Figure 13C,D). A linear fit between anti-p145 and anti-GAS IgG titres gave an R^2^ of 0.62, supporting the usage of p145 to assess the quality of generated antibodies, even in the presence of a *S. aureus*-related immune responses. In addition, the anti-GAS titres correlated with opsonization efficacy against standard GAS strains, using 10-fold diluted sera, in a four-parameter dose–response sigmoidal pattern, gave an R^2^ of 0.9, and 50% inhibitory anti-GAS log_10_ IgG titres (IC_50_) of 2.5 (or 1.5 for undiluted sera) against standard GAS strains, which is equivalent to anti-J8 log_10_ IgG titres of about 4.85 (or 3.85 for undiluted sera).

**Figure 14:**
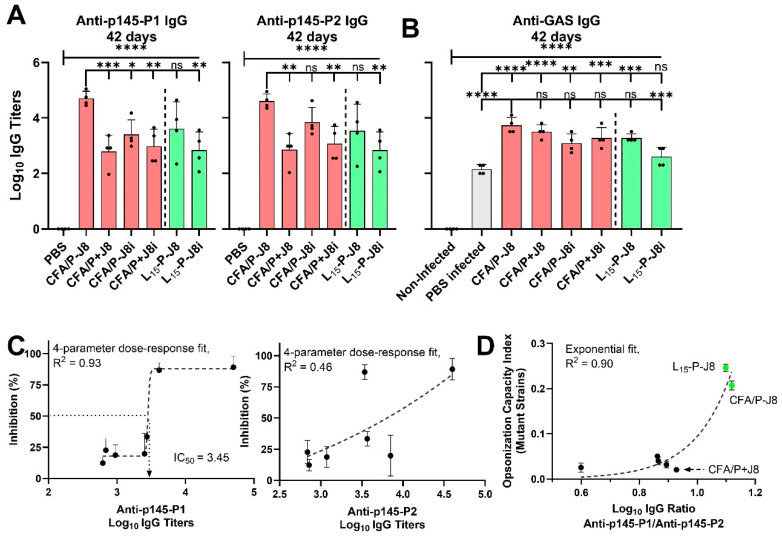
Immune response analyses. (**A**) Serum anti-p145-P1, anti-p145-P2, and anti-GAS_NS1944_ mutant strain using the ELISA. Correlations among the generated immune responses and opsonisation inhibition of mutant GAS strains (NS1944 and NS5582): correlation between opsonization bactericidal activity and (**B**) serum anti-p145-P1 or serum anti-p145-P2 IgG titres, and (**C**) the ratio between serum log_10_ anti-p145-P1 and anti-p145-P2 IgG, where green data points represent the sera of mouse groups immunized with L_15_-P-J8 and CFA/P-J8. Data points were fitted using either a 4-parameter dose–response Boltzmann (panel (**C**)) or an exponential fit (panel (**D**)). One-way ANOVA with Dunnett’s multiple comparisons test was used for statistical analysis. Significance was set to *p* < 0.05 (*), *p* < 0.01 (**), *p* < 0.001 (***), and *p* < 0.0001 (****).

To further investigate whether multiple B-cell epitopes are present within J8i/p145 peptide (LRRDLDA-**SREAKKQVEKAL**-E, bold sequence represents J8i within p145), p145 was synthesized but split into two smaller peptides; the first was 95% conserved in all employed, mutant and standard, GAS strains [p145-p1: RDLDA**SREAK**], and the second was significantly changed, and thus only 30% conserved [p145-p2: **KKQVEKAL**]. The immunized mouse group sera against CFA/P-J8, CFA/P+J8, CFA/P-J8i, CFA/P+J8i, L_15_-P-J8, and L_15_-P-J8i were tested for their IgG antibody titres against p145-P1 and p145-P2 peptides and the inactivated mutant NS1944 GAS strain (Figure 14). The total anti-p145 titres were approximately equal to the sum of anti-p145-P1 and anti-p145-P2 IgG titres (Figure 14A). Thus, IgG titres against the split peptides p145-P1 and p145-P2 support the hypothesis that at least two B-cell epitopes are located within the p145 sequence. Further, the antibodies generated against p145-P1 conserved peptide were well-correlated with opsonic bactericidal inhibition of ‘the mutant NS1944 GAS’ strain; the fit gave R^2^ = 0.93, with anti-p145-P1 IC_50_ corresponding to log_10_ of 3.45, at a 10-fold serum dilution (Figure 14B,C). This suggested that (a) antibodies generated against the p145-P1 conserved peptide fragment are mainly responsible for the opsonic activity against mutant GAS strains, and (b) that mutant GAS strains require the generation of higher log_10_ antibody titres of the high-quality anti-GAS_NS1944_ antibodies for effective opsonic activity (Figure 14B,C) compared to anti-GAS IgG titres required for opsonic activity against standard GAS strains (Figure 12D). To further support this finding, the ratio of anti-p145-P1 to anti-p145-P2 IgG titres was well-correlated with opsonization capacity against the mutant NS1944 GAS strain, normalized by anti-p145 IgG titres (Figure 14D), and ratio values above unity, i.e., vaccines that generated more anti-p145-P1 than anti-p145-P2 IgG titres (CFA/P-J8 and L_15_-P-J8), were highly opsonic against the mutant strains. The ratio of anti-p145-P1 to anti-p145-P2 IgG titres was higher in the vaccines that employed (a) J8 as the B-cell antigen, i.e., GCN-4 sequence flanking of J8i, and (b) conjugation of PADRE to the *N*-terminus of J8. The absence of those two factors focused the generated serum high-quality antibody responses toward the less conserved *C*-terminus portion of the p145 peptide, i.e., p145-P2, and rendered these responses less effective against the mutant GAS strains, such as those generated by CFA/P+J8, CFA/P-J8i, or CFA/P+J8i vaccines (Figure 14D). Thus, we recommend evaluating immune responses against p145-P1 and p145-P2 peptides in future J8-based vaccine development endeavours to assess immune responses against the conserved versus mutant/altered sequences of the p145 peptide. These responses may provide powerful immune correlates of protection against mutant hypervirulent GAS strains.

Although functional antibody responses were demonstrated using in vitro opsonization assays [20,21]—an established correlate of protection—future studies will include live GAS infection challenge studies to assess the overall vaccine-induced protection, bacterial clearance, and survival outcomes. The immunological assays validated here will help optimize and interpret such in vivo challenge studies, particularly in the context of co-infection.

## 5. Conclusions

In sum, P-J8 proved to be a valuable vaccine antigen against both standard and mutant GAS strains with altered p145 sequences in their M-proteins. Conjugation of PADRE T helper to J8 proved valuable to opsonic bactericidal efficacy against mutant GAS strains, while P-J8i proved useful as a vaccine antigen only against standard GAS strains, and conjugation to PADRE was necessary for generating opsonic antibodies. While helicity plays a major role in the activity of M-protein-derived antigens, it is not the sole player in generating high-quality opsonic antibodies against GAS, as our results suggest the presence of at least two B-cell epitopes within the J8i sequence, which are dramatically affected by the neighbouring conjugated peptides, like PADRE and/or flanking GCN-4 sequences, thus significantly impacting their generated antisera’s opsonic activity.

CTD2, NTD, and p145 peptides are of diagnostic value for GAS infection, more appropriate than whole pathogens or whole proteins, which have shared antigens among different bacterial pathogens, and thus may indicate infection by other pathogenic bacteria than GAS. These novel peptide antigens CTD2 and NTD generated significantly opsonic sera, although not as powerful as J8, thus they require further development to impart helicity, e.g., via flanking with GCN-4 and conjugation to a carrier protein, a T-helper cell epitope, or both. Such novel and highly conserved antigens will prove useful if their antisera opsonisation is further enhanced in future studies.

Immunological assays vary in their usability under clinical and ‘advanced’ pre-clinical conditions, and immune sera cannot be evaluated only with anti-GAS ELISA to detect GAS-specific antibody titres if prior infections may have occurred. Thus, testing pre-immunisation sera is important, and conducting ELISAs against GAS-specific peptide or protein antigens could be useful to establish GAS-specific immunogenicity and immune response avidity, respectively. In contrast, the opsonisation assay seems to be less affected by co-infection; however, the interfering antibody titres against shared epitopes were modest, and thus the effect on opsonic efficacy may vary if proper anti-*S. aureus* titres are to develop.

## Figures and Tables

**Table 1:** Sequence of all synthesized peptides.

Peptide	Peptide Sequence	Analytical Data	Purity	Yield
J8	QAEDKVKQSREAKKQVEKALKQLEDKVQ Molecular weight: 3282.6 g/mol.	ESI-MS: [M + 3H]^+3^ *m*/*z* 1095.8 (cal. 1095.3), [M + 4H]^+4^ *m*/*z* 821.0 (cal. 821.5); HPLC, t_R_ = 20.0 min (C18 column, 0–100% solvent B, 53 min)	98%	53%
J8i	SREAKKQVEKAL Molecular weight: 1385.6 g/mol.	ESI-MS: [M + H]^+^ *m*/*z* 1387.9 (cal. 1387.6), [M + 2H]^+2^ *m*/*z* 693.5 (cal. 694.5); HPLC, t_R_ = 15.4 min (C18 column, 0–100% solvent B, 53 min)	97%	55%
J8i-J8i	SREAKKQVEKALSREAKKQVEKAL Molecular weight: 2754.2 g/mol.	ESI-MS: [M + 3H]^+3^ *m*/*z* 920.0 (cal. 919.1), [M + 4H]^+4^ *m*/*z* 690.6 (cal. 689.6), [M + 5H]^+5^ *m*/*z* 552.0 (cal. 551.8); HPLC, t_R_ = 19.4 min (C18 column, 0–100% solvent B, 53 min)	93%	57%
P145	LRRDLDASREAKKQVEKALE Molecular weight: 2354.7 g/mol.	ESI-MS: [M + 2H]^+2^ *m*/*z* 1177.9 (cal. 1178.4), [M + 3H]^+3^ *m*/*z* 785.1 (cal. 785.9), [M + 4H]^+4^ *m*/*z* 588.7 (cal. 589.7); HPLC, t_R_ = 19.3 min (C4 column, 0–100% solvent B, 53 min)	95%	52%
NTD	LRKLKKGTASVAVAL Molecular weight: 1553.9 g/mol.	ESI-MS: [M + 2H]^+2^ *m*/*z* 777.7 (cal. 778.0), [M + 3H]^+3^ *m*/*z* 518.5 (cal. 519.0); HPLC, t_R_ = 18.9 min (C18 column, 0–100% solvent B, 53 min)	96%	55%
CTD2	KALKEQLAKQAEELAKLR Molecular weight: 2066.5 g/mol.	ESI-MS: [M + 2H]^+2^ *m*/*z* 1034.8 (cal. 1034.3), [M + 3H]^+3^ *m*/*z* 685.6 (cal. 689.8); HPLC, t_R_ = 19.9 min (C18 column, 0–100% solvent B, 53 min)	97%	53%
P-J8	AKFVAAWTLKAAA-QAEDKVKQSREAKKQVEKALKQLEDKVQ Molecular weight: 4653.4 g/mol.	ESI-MS: [M + 4H]^+4^ *m*/*z* 1164.8 (cal. 1164.4), [M + 5H]^+5^ *m*/*z* 932.2 (cal. 931.7), [M + 6H]^+6^ *m*/*z*:776.9 (cal. 776.6); HPLC, t_R_ = 21.2 min (C18 column, 0–100% solvent B, 53 min)	96%	43%
P-J8i	AKFVAAWTLKAAA-SREAKKQVEKAL Molecular weight: 2715.2 g/mol.	ESI-MS: [M + 2H]^+2^ *m*/*z* 1357.9 (cal. 1358.6), [M + 3H]^+3^ *m*/*z*:907.0 (cal. 906.1), [M + 4H]^+4^ *m*/*z*:681.4 (cal. 679.8); HPLC, t_R_ = 28.0 min (C18 column, 0–100% solvent B, 53 min)	98%	45%
P-J8i-J8i	AKFVAAWTLKAAA-SREAKKQVEKALSREAKKQVEKAL Molecular weight: 4083.8 g/mol.	ESI-MS: [M + 3H]^+3^ *m*/*z* 1363.1 (cal. 1362.3), [M + 4H]^+4^ *m*/*z*: 1023.1 (cal. 1022.0), [M + 5H]^+5^ *m*/*z*: 818.5 (cal. 817.8); HPLC, t_R_ = 21.9 min (C18 column, 0–100% solvent B, 53 min)	97%	46%
L_15_-P-J8	LLLLLLLLLLLLLLL-AKFVAAWTLKAAA-QAEDKVKQSREAKKQVEKALKQLEDKVQ Molecular weight: 6350.8 g/mol.	ESI-MS: [M + 5H]^+5^ *m*/*z* 1269.8 (cal. 1271.2), [M + 6H]^+6^ *m*/*z*: 1059.7 (cal. 1059.5), [M + 7H]^+7^ *m*/*z*: 908.5 (cal. 908.3); HPLC, t_R_ = 37.2 min (C4 column, 0–100% solvent B, 53 min)	96%	23%
L_15_-P-J8i	LLLLLLLLLLLLLLL-AKFVAAWTLKAAA-SREAKKQVEKAL Molecular weight: 4454.7 g/mol.	ESI-MS: [M + 4H]^+4^ *m*/*z*: 1115.2 (cal. 1114.7), [M + 5H]^+5^ *m*/*z*: 892.5 (cal. 891.9); HPLC, t_R_ = 38.4 min (C4 column, 0–100% solvent B, 53 min)	95%	18%
L_15_-P-J8i-J8i	LLLLLLLLLLLLLLL-AKFVAAWTLKAAA-SREAKKQVEKALSREAKKQVEKAL Molecular weight: 5783.1 g/mol.	ESI-MS: [M + 5H]^+5^ *m*/*z* 1157.2 (cal. 1157.6), [M + 6H]^+6^ *m*/*z*: 964.8 (cal. 964.9), [M + 7H]^+7^ *m*/*z*: 826.9 (cal. 827.2); HPLC, t_R_ = 35.5 min (C4 column, 0–100% solvent B, 53 min)	95%	21%
L_15_-P-p145	LLLLLLLLLLLLLLL-AKFVAAWTLKAAA-LRRDLDASREAKKQVEKALE Molecular weight: 5381.7 g/mol.	ESI-MS: [M + 4H]^+4^ *m*/*z* 1346.8 (cal. 1346.4), [M + 5H]^+5^ *m*/*z*: 1076.5 (cal. 1077.3), [M + 6H]^+6^ *m*/*z*: 897.5 (cal. 898.0); HPLC, t_R_ = 34.2 min (C4 column, 0–100% solvent B, 53 min)	96%	19%

## Data Availability

Data Available upon request.

## Supplementary Materials

The following supporting information can be downloaded at https://www.mdpi.com/article/10.3390/vaccines13060632/s1, Table S1: Quantitative secondary structure estimates of peptide antigens; Figure S1: Capillary electrophoresis analyses examples of pooled faecal samples from infected mouse groups; Figure S2: Pre-immunization serum IgG titers; Figure S3: GAS opsonisation inhibition of non-infected naïve mouse sera and preimmunization infected mouse sera.
