# Supplementary material for: Evaluation of Peptide-Based Vaccines Against Group A Streptococcus in Staphylococcus aureus-Infected Mice
Source: Vaccines (Basel). 2025 Jun 12;13(6):632. doi: 10.3390/vaccines13060632 (PMC12197582; doi:10.3390/vaccines13060632)
Supplement: Supplementary file 1 [file vaccines-13-00632-s001.zip › vaccines-3679840-supplementary.pdf]

vaccines

MDPI

Supporting information

Evaluation of peptide-based vaccines against *Group A Streptococcus* in *S. aureus*-infected mice

Ahmed O. Shalash [1,*], Haolan Sun [1], Yiru Cui [1], Jingwen Wang [1], Barb Arnts [2], Jannah Bauer [2], Waleed M. Hussein [3], Zeinab Khalil [2,3,4], Mariusz Skwarczynski [1], Istvan Toth [1,4]

[1] School of Chemistry and Molecular Biosciences, The University of Queensland, Brisbane, QLD, Australia

[2] Australian Institute for Bioengineering and Nanotechnology, The University of Queensland, Biological Resources, Brisbane, QLD, Australia

[3] Institute for Molecular Bioscience, The University of Queensland, Brisbane, QLD, Australia

[4] School of Pharmacy, The University of Queensland, Brisbane, QLD, Australia

* Correspondence: a.shalash@uq.edu.au

Table S1. Quantitative secondary structure estimates of peptide antigens and vaccines in PBS.

Compounds	Sum	α-Helix %	β-Sheet %	Coil %	Adj. R^2
**PADRE (P)**	2.93999	35.64	0.00	64.36	0.83456
**P-J8i-J8i**	4.90879	22.89	0.00	77.11	0.98149
**P-J8i**	4.77504	25.39	0.00	74.61	0.9089
**P-J8**	5.32364	30.92	0.00	69.08	0.97666
**CTD2**	4.52281	44.27	0.00	55.73	0.90514
**NTD**	5.84523	26.05	24.02	49.93	0.88698
**p145**	4.12839	24.76	0.00	75.24	0.98415
**J8i-J8i**	4.71473	17.25	0.00	82.75	0.98463
**J8**	5.19253	26.74	0.00	73.26	0.96849
**J8i**	3.98814	12.93	0.00	87.07	0.99023

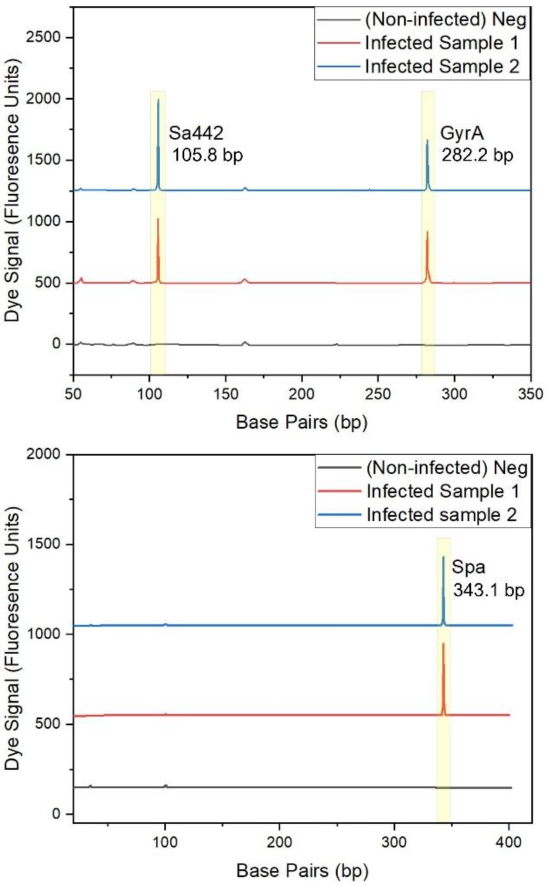

Figure S1. Capillary electrophoresis analyses examples of pooled faecal samples from infected mouse groups compared to non-infected mouse control samples, the highlighted fluorescence peaks on the right represent positive *VS* absent *S. aureus*-specific primers (Sa442, GyrA, and Spa).

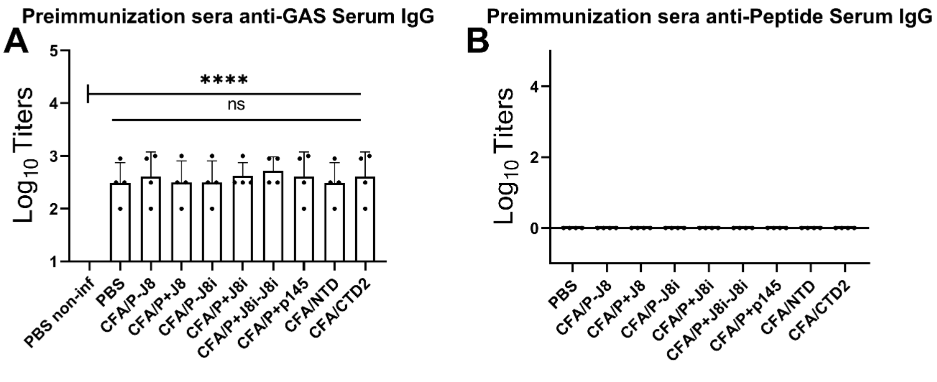

Figure S2. Pre-immunization sera serum IgG titers (A) against GAS, and (B) against the intended peptide vaccine antigens. One-way ANOVA with Dunnett's multiple comparisons test was used for statistics. Significance was set to $p < 0.05$ (∗), $p < 0.01$ (∗∗), $p < 0.001$ (∗∗∗), and $p < 0.0001$ (∗∗∗∗).

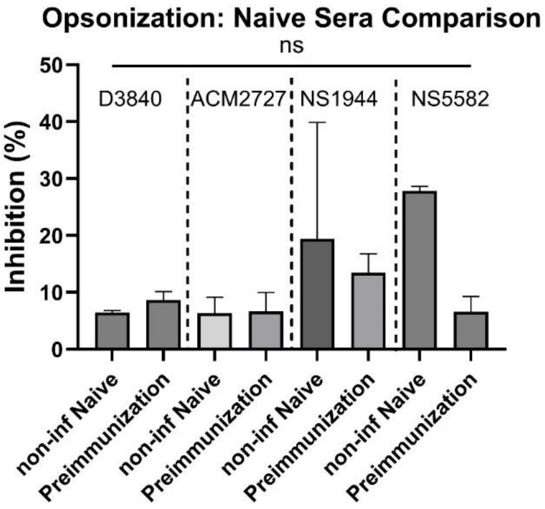

Figure S3. GAS opsonisation inhibition of non-infected naïve mouse sera and preimmunization infected mouse sera against 4 clinical strains. One-way ANOVA with Dunnett's multiple comparisons test was used for statistics, $p > 0.05$ (ns).
